# Multiregion neuronal activity: the forest and the trees

**DOI:** 10.1038/s41583-022-00634-0

**Published:** 2022-10-03

**Authors:** Timothy A. Machado, Isaac V. Kauvar, Karl Deisseroth

**Affiliations:** 1 Department of Bioengineering, Stanford University, Stanford, CA, USA.; 2 Department of Electrical Engineering, Stanford University, Stanford, CA, USA.; 3 Howard Hughes Medical Institute, Stanford University, Stanford, CA, USA.; 4 Department of Psychiatry and Behavioral Sciences, Stanford University, Stanford, CA, USA.

## Abstract

The past decade has witnessed remarkable advances in the simultaneous measurement of neuronal activity across many brain regions, enabling fundamentally new explorations of the brain-spanning cellular dynamics that underlie sensation, cognition and action. These recently developed multiregion recording techniques have provided many experimental opportunities, but thoughtful consideration of methodological trade-offs is necessary, especially regarding field of view, temporal acquisition rate and ability to guarantee cellular resolution. When applied in concert with modern optogenetic and computational tools, multiregion recording has already made possible fundamental biological discoveries — in part via the unprecedented ability to perform unbiased neural activity screens for principles of brain function, spanning dozens of brain areas and from local to global scales.

Improved instrumentation for observing the sky markedly transformed our view of the universe. New tools enabled the detection of subtle and sparse, or massive and universal, interrelated phenomena such as black holes and celestial-body dynamics^1,2^. In neuroscience, we are now experiencing a more rapid but similarly fundamental transformation. Over the past century, we advanced from the invention of tools for measuring electrical activity in single nerve fibres^3^ and neurons^4,5^ to an age when we now use electrical and optical tools to routinely obtain simultaneous, high-speed measurements from thousands of neurons in behaving mammals. But these new methods allow us to do more than just sample from larger numbers of neurons in one brain region. We are now able to record synchronously from large neuronal populations that span multiple brain regions — at, or near, single-cell resolution. This multiregion recording capability offers a burgeoning opportunity to see both the emergent whole and the constituent parts (the forest and the trees) of neural computations in a single dataset. Using these reliable new neurotechnologies, we are poised to make swift progress in understanding how cells in interconnected brain areas work together to produce global brain states and behaviour.

These advances in large-scale neural recording technologies are exciting for the field, but the rapid pace of innovation has made it difficult for researchers to learn about each new approach that might be relevant for their work. No single recording modality is best for all applications, and efforts to build ever better tools mean that the most viable approach one year might change in the next. Depending on the experimental question of interest, researchers must make choices, and accept trade-offs, along multiple different axes, such as spatial resolution, field of view, temporal resolution and cost. Different methods are also differentially compatible with optogenetics, freely moving behaviour and molecular phenotyping approaches. Moreover, there are many approaches to analysing and deriving insight from the resulting datasets, with each analysis method offering different perspectives and biases.

Here we summarize advances in multiregion recording and analysis techniques, consider trade-offs among different technical approaches and review key findings resulting from application to neural coding and brain-wide computation. Other recent reviews have focused on complementary issues, such as optical methods specifically^6,7^, or on conceptual insights that have emerged from the analysis of neuronal population recordings of ever increasing size^8^. In contrast, here we specifically focus primarily on technology and discoveries relating to large multiregion neural datasets, where simultaneous recordings were obtained from neuronal populations spanning many areas of the brain. We pay particular attention to the rodent literature, which has seen the most dramatic development in this regard in recent years. However, many of the ideas and technologies we discuss are either currently or well on their way to being applied to non-human^9–11^ and human^12^ primates.

## Why do we care about multiregion recording?

Brains are highly interconnected systems, composed of networks of neurons that span spatially distant regions. Anatomical tracing has identified consistent and diverse brainwide connectivity patterns^13^, with individual or neighbouring neurons receiving input from or sending output to multiple regions in parallel (for example, in the primary visual cortex^14^). Research across many decades identified key behavioural and sensory correlates of neuronal activity in particular brain regions, leading to the assignment of primary functions to these regions. However, until the past few decades, most recording of neuronal activity was limited to monitoring a handful of neurons in one region at a time — a situation that has changed dramatically with the development and application of new methods^15–17^. Because of the interconnectivity and nonlinearity of neural circuits, it is not guaranteed that the conclusions reached by observing a few neurons in one region will also apply to data taken from neurons across many regions. Thus, many questions have persisted, and others have newly emerged, about the extent to which individual brain regions perform individual functions, whether specific computations are local or distributed, and whether brain states and representations are broadcast or confined.

As researchers began to obtain population recording data from multiple brain regions simultaneously, behaviourally relevant neuronal codes were found to be distributed across the brain^18–20^. For example, motor actions were found to modulate neural activity in many non-motor areas — including in the sensory cortex^21–24^. Overall, an intriguing picture of planning and outcome processing is beginning to emerge, in which neural computations are distributed, information is distributed or both. Local computations appear to be important, but also should perhaps not be analysed in isolation. We now must investigate why representations of sensation, cognition and action are so widespread, and what role they play in guiding behaviour^20^. In the past decade, much work has shifted from a focus on the computational properties of single neurons to a population doctrine that is focused on the computations performed by groups of neurons from a given brain region^25^. In an analogous fashion, as population recording methods continue to scale up from single to multiple regions, perhaps a comparable shift in perspective will emerge from the study of multiregion population dynamics.

## A common taxonomy of brain regions

To synthesize findings about multiregion neural dynamics across studies, a common taxonomy of brain regions (or areas; here either word is used interchangeably) is required. Over the past few decades, a small number of rodent brain atlases have become widely adopted^26–28^. Until recently, atlas borders between areas were primarily delineated on the basis of cytoarchitectural differences (that is, clear differences in the particular arrangement and density of neurons between regions). More recently, a wealth of additional information has become available to enumerate and delineate brain regions, including viral-based connectivity^13,29^ and gene expression patterns^30,31^. These disparate data streams have been combined into an updated 3D atlas: the Allen Mouse Brain Common Coordinate Framework version 3 (CCFv3)^32^. For electrophysiology data, either 2D sections or 3D volumes can be obtained post hoc from fixed experimental brains so as to reconstruct the trajectories of dye tracks left behind by recording electrodes^18,33,34^, unstained tracks visible from larger probes^35,36^ or tissue damage left by electrolytic lesions^37,38^. For optical data, alignment can be achieved using known anatomical or functional landmarks^23,39^. As a whole, it is encouraging to see that increasingly more rodent studies are aligning their functional brain data to a common reference atlas; this trend is likely to continue along with greater data sharing between research groups. The use of a standard anatomical framework such as CCFv3 may also permit the development of searchable databases that use atlas-registered physiology data to generate summary statistics and perform meta-analyses across thousands of published articles — as has been done for the human neuroimaging community with tools such as NeuroQuery and Neurosynth^40,41^.

## Recording techniques

Three main technical approaches are currently used to record multiregion neuronal activity: one-photon fluorescence imaging, multiphoton fluorescence imaging and electrophysiology. Optical techniques enable cell type-specific recording by leveraging genetically encoded fluorescent activity indicators such as Ca^2+^ or voltage sensors; cell types are commonly targeted on the basis of genetic expression profiles or by cellular connectivity^42–44^ or assigned on the basis of post hoc registration to cell type-specific labelling^45–47^. Some intact-skull optical techniques also have the potential to be minimally invasive and require no craniotomy — if used in tandem with transgenic animals or intravenous gene transfer to drive sensor expression^48,49^.

**Fig. 1 |:**
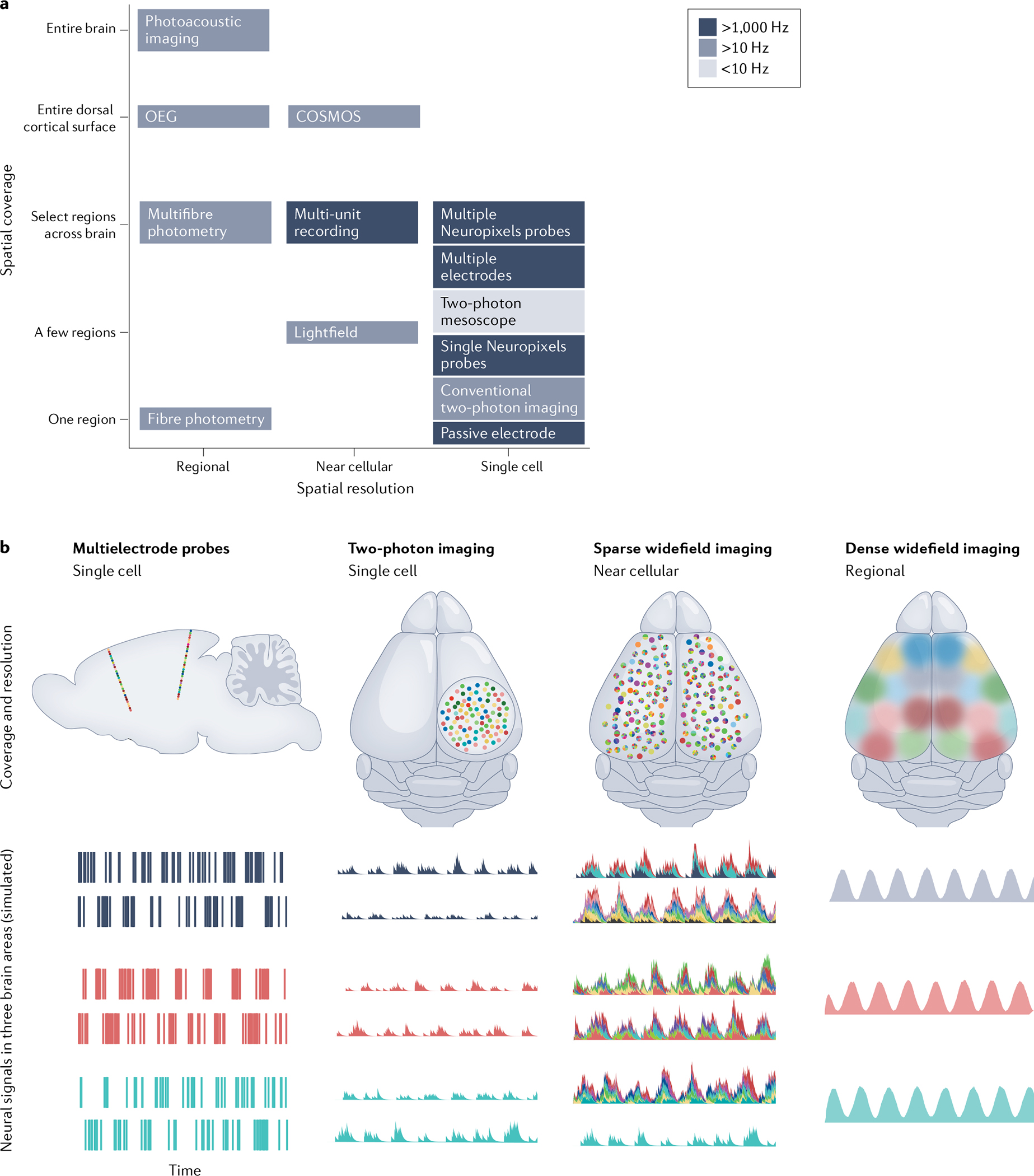
A spectrum of methods for multiregion recording. **a** | Each multiregion recording method exhibits a set of trade-offs among spatial resolution (x-axis; ranging from regional-scale resolution to single-cell resolution), spatial coverage of a simultaneous recording (y-axis; ranging from just one region to the entire brain) and acquisition speed shading. **b** | As a consequence of the trade-offs illustrated in part **a**, the spatial and temporal features of the data diverge between different methods. Synthetic data, simulated on the basis of the characteristics of each method, qualitatively illustrate the kinds of data that are produced by different multiregion recording methods — ranging from sparse sampling of neurons around an electrode array (left panel, top) with high-fidelity single-neuron recordings (left panel, bottom), to complete coverage of dorsal cortex (right panel, top) without full cellular resolution (right panel, bottom). Scales on the synthetic neural activity traces are arbitrary. COSMOS, cortical observation by synchronous multifocal optical sampling; OEG, optoencephalography.

Electrophysiology, on the other hand, offers direct recording of cellular action potentials, at sub-millisecond temporal resolution. This high sampling rate is necessary for resolving the shape and timing of individual action potential waveforms. Additionally, electrophysiology is label-free, and therefore does not require any genetic manipulation of the animal, but also does not readily allow detailed anatomical or molecular understanding of the recorded cells. While it is possible, in some situations, to pair electrophysiology with optogenetics to identify specific types of neurons (‘optical tagging’)^36,50–53^, this approach is of low throughput relative to two-photon imaging, which permits exhaustive characterization of a genetically defined cell type in a local area. Another limitation of extracellular electrophysiology is that, despite recent progress^54–57^, it remains difficult to track recorded single neurons across multiple days with electrophysiology, in contrast to optical methods with genetically encoded fluorescent indicators, where such an approach is relatively straightforward. However, unlike with most optical imaging approaches that require head fixation, or are limited by the potential photobleaching of a fluorescent sensor, it is possible to obtain continuous electrophysiological recordings for many days^57^. In this section, we summarize the current state of diverse brainwide recording methods and discuss their strengths and weaknesses for studying multiregion neural computation (FIG. 1 and TABLE 1).

### One-photon fluorescence

#### Regional-scale widefield imaging of the cortex.

One-photon optical fluorescence techniques use short-wavelength (for example, blue) excitation light to elicit fluorescence of a longer wavelength (for example, green). This process is efficient and enables illumination and imaging of an entire field of view, yielding fast recording speeds that can take advantage of modern scientific CMOS image sensors; these sensors can have acquisition rates of hundreds of frames per second with low read noise (less than one electron) and extremely high sensitivity (quantum efficiencies greater than 0.9). One-photon fluorescence is also relatively robust to illumination alignment and detection parameters, and such techniques are therefore easier and cheaper to implement than alternatives such as two-photon techniques — especially over large fields of view. The primary drawback of one-photon illumination is that any fluorophore in the specimen can fluoresce if it absorbs an excitation photon. This effect can lead to out-of-focus background fluorescence that adds noise to the signal measured at the focal plane (originating from fluorescent molecules in the surrounding tissue).

The development of bright genetically encoded fluorescent activity sensors led to the use of widefield one-photon imaging for simultaneously measuring neural activity across multiple regions of the rodent dorsal cortex^58^. These widefield techniques that combine fast, mesoscopic resolution on the scale of multiple subregions of the brain (even beyond just the cerebral cortex) can be termed ‘optoencephalography’ (OEG), as they offer a perspective reminiscent of electroencephalography, but with the genetic and spatial specificity of optical activity sensors. Early optoencephalographic approaches used synthetic voltage-sensitive dyes applied to exposed cortex^16,59,60^. More recently, the development of transgenic mice that express sensitive and bright genetically encoded Ca^2+^ sensors (for example, GCaMP) emerged as a reliable means of obtaining cell type-specific recordings from across the cortex^61–63^. Additionally, the development of viral capsids that efficiently cross the blood-brain barrier enabled intravenous delivery of desired transgenes (for example, encoding GCaMP) across the brain^48,49,64^. Of practical importance, the skull can even be made sufficiently transparent to facilitate widefield imaging with no craniotomy, through refractive-index matching by application of optical glue or cement^65^. However, due to scattering (as well as typically dense neuronal labelling), regional-scale optoencephalographic approaches should be considered as mesoscopic, with information in a typical pixel derived from regional activity on the order of thousands of neurons.

Microscopes suitable for OEG must offer high light collection with good image quality across a large field of view, particularly when imaging more-sensitive but less bright fluorophores such as GCaMP6f. A common approach is inspired by a tandem-lens design with consumer camera lenses that were originally used for intrinsic imaging^66^. One key consideration is that the optoencephalographic signal can be contaminated by time-varying haemodynamic artefacts, caused by variation in absorption as the amounts of oxygenated and deoxygenated haemoglobin fluctuate in a given area of the brain. This artefact can be corrected by measuring the Ca^2^+-independent fluctuations, either by using the isosbestic excitation wavelength of GCaMP around 410 nm (REFS.^48,67^) or by measuring haemodynamic absorbance with reflected green light^63,68^, and subtracting this from the raw Ca^2^+-dependent optoencephalographic signal. Failing to correct for haemodynamic artefacts may lead to spurious conclusions and will hinder reproducibility both within and between mice.

Precisely what is being measured by an optoencephalographic imaging technique depends on the specific experimental preparation — and the means of delivering the fluorescent sensor. Specific cell types are commonly targeted using genetically or anatomically delivered recombinases such as Cre, which through recombination enable cell type-specific expression of an indicator gene that is more universally present but in a recombinase-dependent form. Many relevant Cre-driver transgenic rodent lines have been created^62,69,70^, including as part of the BRAIN Initiative Cell Census Network^71,72^, along with diverse viral vectors carrying genetically encoded indicators that can even depend on two or three different recombinases for highly specific expression^44,73^. Targeted cell types can be excitatory (for example, VGLUT1 expressing), inhibitory (GAD2 expressing, somatostatin expressing or parvalbumin expressing) or cortical layer specific. Drivers exist for layer 2/3 *(Cux2*–*Cre),* layer 4 *(Scnn1a*–*Tg3-Cre),* layer 5 *(Rbp4*–*Cre)* and layer 6 *(Ntsrl*–Cre)^74^. With less specific expression, for example when an *Slc17a7*–*Cre* mouse is used to drive GCaMP expression across all cortical layers, Monte Carlo simulations have suggested that most of the signal should arise from layer 2/3 (REF^75^), although this may depend on the specific expression pattern, and there is evidence that most of the optoencephalographic signal derives from fluorescence emitted by layer 1 neuropil that may include layer 5 dendrites^48^. The combinatorial use of different sensors also affords new experimental opportunities. For example, two-colour optoencephalographic imaging has been used to record from excitatory and inhibitory populations^48^; one recent implementation combined a red fluorescent Ca^2+^ indicator (jRCaMP1b) with a green fluorescent acetylcholine sensor (ACh3.0)^76^. Beyond Ca^2^+ sensors, other genetically encoded fluorescent sensors can be used in conjunction with these same optical methods and Cre lines to enable the brainwide measurement of the release of glutamate, GABA or dopamine^77,78^. Finally, a new generation of voltage sensors is becoming available that may be more suitable than Ca^2+^ sensors for some widefield imaging experiments (see ‘Voltage imaging’).

Regarding the use of transgenic animals to express genetically encoded sensors, it is important to be aware that the expression of any non-native protein in the brain (especially during development) may lead to changes in cellular, or even circuit-level, function. For instance, it has been shown that using some strains of transgenic mice to express the Ca^2+^ sensor GCaMP during development can lead to aberrant cortical activity (reminiscent of seizures) in some mouse lines^79^. Therefore, it is always important to validate an experimental preparation by performing appropriate control experiments. In this specific case, use of inducible GCaMP lines, where the expression of non-native protein can be delayed until mice have reached adulthood, can mitigate this issue^79^.

Beyond the cortex, OEG has been successfully applied to other structures along the surface of the brain — namely the cerebellum^80^ and superior colliculus^81,82^. Moving forward, it will be exciting to develop new experimental preparations that will enable simultaneous visualization of these structures in addition to the surface of the dorsal cortex. OEG has also recently been extended to freely moving settings, with head-mounted microscope designs for rats^83^ and mice^84^. Finally, OEG can be paired with other techniques, such as whole-brain functional MRI^85^, or use of home-cage systems where mice learn to head-fix themselves for widefield imaging^86,87^.

#### Cellular-scale widefield imaging of the cortex.

A number of steps are required to advance beyond the regional-scale (millimetre scale in the mouse) spatial resolution of OEG. First, the quality of optical access to the brain must be improved beyond that afforded by an index-matched clear skull preparation. Among other steps, this improvement requires a large craniotomy, as well as a clear window that is curved to match the curvature of the brain, and made from glass^88^ or plastic^89^. It is possible to flatten the brain to some extent, but there is a limit to how large the window can be before significant tissue damage is caused^48^. Craniotomies have been successfully demonstrated with use of manual surgical techniques or with the semi-automated assistance of a robotic stereotaxic apparatus^23^.

Second, to address defocused signal and scattering, fluorescent protein expression must be restricted, either to a subset of neurons or to a localized part of each neuron. In one approach, the use of tamoxifen-dependent, layer 2/3/4-restricted expression of *Cux2*–*CreER* allowed limitation of GCaMP expression to a sparse set of superficial neurons^23^. The influence of layer 1 neuropil signal was thereby minimized (relative to less specific expression strategies used for widefield imaging). Other strategies include driving sparse expression using intravascular injections of blood–brain barrier-crossing adeno-associated virus variants such as PHPeB^49^. Limiting neuropil fluorescence can also be helpful in this respect, most efficaciously and definitively through the use of nuclear-restricted GCaMPs created by histone H2B fusions^90–92^, although other forms of targeting can include partial restriction of GCaMP to cell bodies through use of peptide tags derived from potassium channels (Kv2.1) or ribosomal subunits^93–95^.

Third, an imaging system is needed to permit recording across the curved, centimetre-scale extent of the mouse dorsal brain surface, with high light collection and good image quality. For mesoscopic OEG, the curvature of the brain surface is less of an issue because the defocus blur is itself roughly on par with the spatial resolution of the technique. When the goal is cellular or near cellular resolution, however, the defocus becomes a severe limitation on the accessible field of view^88^. This is due to the curvature of the dorsal surface of the brain, and is therefore an issue regardless of whether the dorsal skull has been replaced with a curved-glass window^23,88^ or a polymer-based window^89,96^.

To address this issue, cortical observation by synchronous multifocal optical sampling (COSMOS) uses a bifocal lenslet array and a single camera sensor to simultaneously record in-focus videos of the medial and lateral regions of the cortical surface. This approach has been used to record ~30 Hz signals from thousands of cellular to near cellular resolution neuronal sources simultaneously across the entirety of the mouse dorsal cortex^23^. A more complex technique (real-time, ultra-large-scale, high-resolution imaging) uses a set of 35 cameras arranged in a 5 × 7 array and a custom objective lens to achieve gigapixel imaging of neuronal dynamics across the curved cortical surface^97^. Additional possible tactics include use of fast tunable lenses, although this approach is hindered by optical aberrations and a trade-off between the speed of tunability and the size of the optical aperture. By combining high numerical aperture objectives with high-resolution cameras, lightfield^98,99^ and light-sheet^100–102^ microscopes can potentially enable truly volumetric multiregion imaging; moreover, eventually these approaches may be miniaturized to the level of applicability in freely behaving rodents. Along these lines, the Computational Miniature Mesoscope used miniaturized lenslet optics in a first step towards head-mounted, cortex-wide, volumetric imaging in freely moving rodents, although considerable development work will still be required to achieve that goal^103^.

One-photon imaging techniques in scattering mammalian tissue do not guarantee true single-cell resolution. For example, with use of a visual-stimulus assay with comparison with ground-truth high-magnification two-photon data, different neuronal sources computationally extracted from COSMOS data were estimated to be derived from 1–15 neurons^23^. Similar preparations using different Cre lines, or higher-magnification objectives, may yield results even closer to single-cell resolution^104^, but all such one-photon microscopy approaches fundamentally lack the axial resolution to guarantee single-neuron resolution. Still, it has been demonstrated that these cellular-scale data occupy a fundamentally different regime of experimental utility, compared with much lower resolution regional-scale widefield approaches. Although one-photon cellular-scale recording techniques should not be generally used to make claims about the response properties of individual cells, these methods permit the study of high-dimensional population coding across large neuronal ensembles^23^.

#### Multifibre photometry.

While OEG and COSMOS provide straightforward access to multiple superficial brain areas, these optical approaches are not readily applicable for imaging deep regions. Therefore, to reach areas deep in the brain, it is common to remove tissue or implant a light conduit, taking advantage of the fact that one-photon illumination is easy to transport through a multimode optical fibre. Such fibre photometry^105^ techniques can be sensitive enough to acquire activity signals arising from axons deep in the living mouse brain, while also being compatible with optogenetics, and enable cell type-specific optical recording access to anywhere in the brain that can be reached by an implanted fibre optic cannula^105–109^, although they average signal across many neurons in a volume^110^. Similar principles have been adapted for recording using genetically encoded voltage sensors^111^, and use of a tapered fibre can even enable depth-resolved recording from along the extent of the fibre^112^. Importantly, this fibre-based recording approach can be extended to multiple implanted fibres to enable multiregion recording, as demonstrated by frame-projected independent-fibre photometry^67^, as well as by a large-scale photometry technique that uses high-density arrays of optical fibres to simultaneously target up to 48 brain regions^113^. Of course, increasing the number of optical fibres inserted into the brain displaces more brain tissue — with greater potential for adverse effects on circuit function and behaviour, a theme common to all forms of brain interfacing, including electrophysiology and microendoscopy (which requires implanted lenses). In each case, the size and the number of neural implants must be balanced against concerns about potential damage; validating relevant baseline behaviour of animal subjects is always important in this regard.

#### Voltage imaging.

With extracellular electrophysiological recordings (discussed later), it is difficult to know what fraction of the active neurons surrounding an electrode are being sampled — especially when there are many sparsely firing cells. Identifying spatial structure at fine spatial scales is also difficult: electrode arrays do not permit precise localization of units except in very limited scenarios (for example, 2D arrays on organotypic slices or cell cultures). On the other hand, Ca^2+^ imaging presents different limitations. Ca^2^+ sensors permit high spatial resolution but provide an indirect, and low-pass-filtered, measure of action potential firing^114–116^ (also see BOX 1). While models exist to estimate action potential firing rates from Ca^2+^ measurements, in some situations it would be preferable to simply measure voltage directly. Indeed, cellular resolution, high-speed voltage imaging techniques could represent the best of both worlds — offering genetic specificity in recordings, dense measurements from even sparsely active neurons, an optical readout of action potential waveform shape and even information about subthreshold membrane voltage^115,117–121^.

Significant progress has been made over the past decade along these dimensions. A host of novel genetically encoded voltage indicators are now available, including the ASAP family^119,122^, ArcLight^118^, the QuasAr family^121,123–127^ and Voltron (which requires the addition of a synthetic Janelia Fluor fluorophore)^117^. With use of the latest variants of these tools, it is now possible to perform cellular resolution voltage imaging such that both action potentials and subthreshold signals can be measured in single trials, from small ensembles of neurons. Variants of QuasAr are also compatible with optogenetic tools such as CheRiff (a blue-shifted channelrhodopsin) or other newly developed red-shifted opsins such as ChRmine^123,125,126,128^; this approach could lead to the voltage sensor version of all-optical reading and writing of neural activity into neural ensembles to modulate animal behaviour, as achieved with Ca^2+^ sensors previously.

Together, these results point to a promising future for voltage imaging. Unfortunately, at the moment, there are also significant challenges that must be overcome before Ca^2^+ imaging or electrophysiology is displaced — especially for multiregion experiments. First, voltage dynamics are much faster than Ca^2+^ dynamics, necessitating that high signal-to-noise ratio (SNR) optical signals be measured at kilohertz rates to resolve action potential waveforms, compared with the 2–200-Hz range seen with Ca^2+^ imaging data. Consequently, genetically encoded voltage indicators must emit far more photons per unit time than Ca^2+^ sensors to achieve a comparable SNR, because signals can be integrated for far less time per frame. To remedy this issue, most voltage imaging systems use one-photon methods to image small fields of view. The most obvious alternative would be to increase the excitation laser power to levels that might damage tissues of interest, or to use two-photon methods that require novel approaches for fast laser scanning, such as beam multiplexing.

In line with this, recent articles have presented two-photon microscopy approaches called ‘ultrafast local volume excitation’ and ‘free-space angular chirp-enhanced delay, which were reported to permit in vivo measurement of action potentials and subthreshold dynamics with the ASAP3 genetically encoded voltage indicators (but only from 3 and 20 simultaneously recorded neurons, respectively^122,129^). One-photon approaches can measure activity from more neurons at coarser spatial resolution — but due to constraints of camera acquisition rate, thermal damage and photon-flux concerns, only up to a few dozen neurons can be imaged simultaneously^117,120,123^. True multiregion population voltage data are, at the moment, attainable only by combining use of the existing genetically encoded voltage indicators with imaging methods that lack cellular resolution, such as OEG and fibre photometry^111,130,131^. But a recent preprint reports integration of a custom two-photon system, a new voltage sensor (SpikeyGi) and a nonlinear denoising algorithm (DeepVid) that permitted in vivo imaging of approximately 100 neurons for over 1 h (REF.^132^). While this approach remains to be validated, especially because denoising algorithms rely on difficult-to-characterize supervised deep learning methods, this progress gives reason to believe that within a few years population voltage imaging may become more broadly applicable — at least for measuring single-region neural population activity.

#### Photoacoustic imaging.

Another approach to brainwide recording of neural signals (versus haemodynamic or structural signals) uses the optoacoustic effect. Here, ultrasound waves are generated by transient light absorption, which can be detected through centimetres of tissue. By the pulsing of bright one-photon excitation, changes in fluorescent indicator absorbance can be measured throughout the entire brain volume^133^. Unlike functional MRI or intrinsic imaging methods which measure haemodynamic signals, photoacoustic methods using genetically expressed fluorescent indicators directly measure signals emitted from neurons. Ultrasound transducer arrays must be coupled to the brain by water or gel — as with high-resolution optical microscopy methods that rely on immersion objective lenses. But to enable whole-brain tomography, these ultrasound arrays must be coupled over a much larger area. These steric constraints may pose a challenge for application of this method to freely moving rodent preparations, and potentially to even some awake-behaving scenarios. In vivo, photoacoustic methods are also limited by the degree to which blue excitation for GCaMP can travel through the brain without blood absorption. However, application of this technique with red-shifted indicators should increase the depth, and will reduce the impact of haemodynamic-related signals. This approach can be combined with the simultaneous use of other ultrasound-based methods for functional stimulation or haemodynamic recording^134^.

### Two-photon fluorescence

While one-photon methods are straightforward to implement and use, are relatively inexpensive and permit video-rate acquisition from molecularly defined neuronal populations across large fields of view, they generally do not offer unambiguous single-cell resolution in scattering mammalian brain tissue. In contrast, two-photon optical fluorescence techniques use very high intensity excitation light of a longer wavelength (that is, infrared) to elicit fluorescence of a shorter wavelength (that is, green). Two-photon fluorescence depends on the square of the excitation light intensity, because sufficient photon density is required to achieve simultaneous fluorophore excitation by two lower-energy photons. This nonlinear dependence affords two key advantages: optical sectioning, which results from restriction of emission to the focal plane, and robustness to scattering, which results from the increased scattering length of infrared light, as well as the raster-scanned photon counting imaging process^135^. Disadvantages of two-photon methods include the high cost of the pulsed laser and inherent speed limitations of a raster-scanned approach.

Originally, two-photon microscopy approaches used high-magnification objectives to observe sub-millimetre fields of view. Over the past few years, there have been efforts to extend this technique to record from multiple regions. One approach is to use two high-magnification objective lenses, with separate beam paths^136^. With careful planning, these objective lenses can be positioned across the brain, potentially in tandem with implanted endoscopes or with optogenetic manipulation of additional regions^137^.

Another approach is to use a single, large, low-magnification objective lens. Because of the specialized high numerical aperture requirements of two-photon imaging, this approach has required the design of expensive, customized objective lenses. The two-photon random access mesoscope developed by Sofroniew et al. has a 5-mm-diameter field of view, with a numerical aperture of 0.6, near-diffraction-limited performance and a remote focusing module to allow access to multiple focal planes across the imaged volume^138^. The raster-scan pattern is adjustable, and can image the entire field of view at up to 4.3 frames per second at low resolution or 0.7 frames per second at high resolution.

Higher speeds can be achieved by imaging a few subregions, yielding performance similar to that of multiple objective lens microscopes. The Trepan2p microscope has a 3.5-mm-diameter field of view, with a numerical aperture of 0.43 NA, diffraction-limited performance with a curved field and a tunable lens for volumetric acquisition^139^. The full field of view could be scanned with one beam at 0.1 frames per second, but the microscope has two separate beam paths to enable simultaneous acquisition of two smaller subregions at 30 frames per second.

Other microscopes have been explicitly designed to image multiple subregions with one objective, for scenarios wherein it would be mechanically difficult to place two objective lenses next to each other, such as when one is imaging the primary and secondary somatosensory cortex^140,141^. The Diesel2p mesoscope has a 5-mm-diameter field of view, with a numerical aperture of 0.54, and dual independent scan engines for simultaneous imaging of two regions, or from four regions in the Quadroscope version of the microscope^142,143^. By use of an elongated point spread function, it is possible to scan an entire 4 mm× 4 mm × 100 μm volume, as opposed to just a single focal plane, at 3.2 Hz (REF^144^). Last, light beads microscopy uses a set of axially separate and temporally distinct foci to record nearly simultaneously from the entire axial imaging range, recording from approximately 5.4 × 6 × 0.5 mm^3^ volumes at around 2 Hz — potentially enabling cellular resolution recordings from up to one million total neurons^145^.

Many approaches further increase imaging speed by multiplexing the two-photon beam into many beam-lets that can be scanned in parallel (or remain statically parked on neurons of interest). For example, one microscope with 16 beams and 16 detectors can sample from a 2 × 2 mm^2^ field of view at up to 17.5 Hz (REF^146^), while another uses 400 beams with scientific CMOS camera detection to sample a sub-millimetre field of view at kilohertz frame rates^147^. With two-photon excitation, however, the SNR is reduced for a given laser power due to the focusing of illumination into additional focal spots^148^. Additionally, scattering-related advantages of two-photon imaging begin to decrease as more focal spots illuminate the specimen.

Two-photon imaging in a small field of view has also been combined with simultaneous OEG, through the use of a prism to enable high-magnification access with relatively little obstruction of the OEG field of view^149^. Recently developed head-mounted two-photon microscopes suitable for studying freely moving behaviour in mice^150^ might be productively integrated with OEG to provide broader functional information. High-resolution structural two-photon scans could also be obtained from these same mice under head fixation. Such a multimodal approach would enable registration of freely moving population activity datasets to detailed anatomical and molecular datasets — all at cellular resolution.

### Electrophysiology

Unlike optical methods, extracellular electrophysiology directly records electrical activity associated with action potentials on the millisecond timescale of individual spikes; such high acquisition rates facilitate assigning individual spikes to specific neurons, or units, on the basis of the characteristic shape of each neuron’s spike waveform. However, algorithms to perform this task of ‘spike sorting’ are imperfect, require some manual curation and are sensitive to artefacts from animal movement and probe location drift over time. In recent years, advances have been made towards improving and automating these data-processing techniques, but challenges remain (see BOX 1). Other challenges inherent to electrophysiology, compared with imaging, include reduced compatibility with targeting the readout to genetically or anatomically defined cell types, reduced long-term stability of single-cell identification across days and, until recently, recording simultaneously from only a handful of neurons in vivo. However, with the development of high-density, multiple-site electrodes, it has now become possible to simultaneously record from thousands of units, spanning many brain regions (with straightforward access to subcortical regions in mouse), including during optogenetic control^18,34,56,151^. These advances have been driven primarily by the transition from microwire-based recording systems to silicon-based and polymer-based probes. Microwire tetrode arrays remain a benchmark tool for obtaining stable recordings from single units over many weeks^152^, but this may change over the next decade as easier-to-manufacture silicon-based and polymer-based probes become available.

The choice to use electrophysiological versus imaging methods presents a number of trade-offs^115^. For instance, imaging permits dense sampling of neurons along individual planes, whereas electrode-based methods sparsely sample neurons along the depth of each recording probe^56,153^, or from dispersed points in space where tetrodes have been placed^17,152^. For a discussion of these considerations and others regarding temporal resolution, spatial sampling, and optogenetic compatibility, see BOX 2.

#### Silicon-shank probes.

Silicon-based probes with tens to thousands of electrical contacts per shank are now widely available — a major increase versus microwire arrays, which typically consist of a few dozen wires at most^152^. Silicon-shank probes are also significantly narrower than microwire-based probes, therefore reducing tissue damage. More recently, silicon-based probes (termed Neuropixels) were developed with active amplification and digitization on the base of each probe itself^151^. This design significantly increases the SNR, especially in freely moving settings. These Neuropixels 1.0 probes are manufactured using CMOS nanofabrication and, in their most common configuration, have 960 recording sites across an ~4-mm linear span, with up to 384 sites recordable simultaneously. A single probe thus allows sampling from multiple brain regions, depending on the trajectory of insertion. Multiple successive probe insertions can be used to accumulate asynchronously recorded data from many regions across multiple sessions^18,34^.

More recently, Neuropixels 2.0 probes^56^ were described with a geometry similar to in the original probes, but are also available in a four-shank configuration. This means 384 simultaneous channels can now be measured over an area 750 μm wide and to a depth of 720 μm (with the four shanks evenly spaced across this area). A single headstage can also now mount two probes, permitting another four shanks to be inserted within a few hundred microns of the first probe — with spacing limited only by the mounting fixture used, as the probes themselves connect to the headstages with flexible connectors. Building upon previous work with the first-generation probes^54,151,154^, Neuropixels 2.0 probes are more suitable for long-term (chronic) implantation and for use with freely moving animals owing to their reduced weight (the total weight of two 2.0 probes and a headstage is ~1.1 g versus ~1.3 g for a single 1.0 probe and a headstage; implant weights exclude the weight of the structural materials and cement that must be used to stabilize each probe) and new methods correcting for motion artefacts in acquired data^56,153^.

Use of multiple probes simultaneously, typically in a head-fixed configuration, permits a substantial increase in the number of regions that can be monitored. Recordings in mice have been performed from up to eight simultaneously inserted Neuropixels probes across the brain^22^, or in a targeted manner to investigate visual cortical and thalamic regions^33^. Typically, electrode probes are dipped in lipophilic dyes^155^ so that the insertion track of each probe can be identified in histological sections, or in three-dimensionally cleared tissue^18,33,34^, and are then aligned with a reference atlas such as the Allen Mouse Brain CCFv3 (discussed earlier). But limitations remain, including with respect to compatibility with cell type-specific recording methods such as OEG, since silicon-based probes and headstages are not optically transparent. Recording stability and quality also significantly degrade over time (1–2 months), probably as a consequence of issues with biocompatibility and mechanical damage to the surrounding tissue arising from a lack of flexibility.

#### Flexible polymer-based probes.

In terms of combining multielectrode recordings with OEG or two-photon imaging, flexible or transparent probes may be useful. These properties can be obtained through the development of neural interfaces fabricated on polymer substrates, instead of shanks made of silicon^156–158^. Polymer-based probes such as the Neuro-FITM probe may be particularly useful for simultaneous imaging^158^, which is possible but difficult with silicon-based probes^159,160^. The Neuro-FITM probe is a 32-channel or 64-channel device with electrodes deposited on a flexible polymer, while maintaining a spike SNR comparable with that of Neuropixels probes, and is optically transparent so as to permit simultaneous OEG.

Flexible polymer-based probes may also be valuable for obtaining stable, high-yield recording over many months^57,157^. Again, obtaining such data is possible with modern silicon-based probes^55,56,154^ and tetrode arrays^152^. However, improved biocompatibility relative to microwire bundles and silicon devices may permit increased long-term unit yield, and the flexible nature of polymer-based probes may also be more suitable for recording from larger animals or deep neural structures (such as the brainstem and spinal cord), where the ability of these probes to move and bend with neural tissue compares favourably with the properties of silicon-based probes^57,158,161^. But for now, the capability of modern silicon-based probes such as Neuropixels 2.0 is quite impressive, and it remains to be seen whether alternative, polymer-based approaches will be widely adopted beyond specific use cases, such as where a transparent or highly flexible probe is required.

## Future advances in multiregion recording

Recording methods continue to improve but eventually will encounter physical limits. Will it ever be possible to simultaneously record the action potential firing of every individual neuron in an entire mammalian brain? Theoretical analysis suggests arrays of advanced electrode probes may someday be able to record from most of the neurons in the cortex, or potentially a large fraction of a rodent or primate brain^161^. Nearer term, the highest-yield recording methods are still based on optical imaging techniques with significant trade-offs between spatial and temporal resolution, such as light beads microscopy, which might simultaneously obtain Ca^2+^ signals from up to one million neurons but at ~2 Hz (REF^145^). Multiple optical paths with identical optics could potentially be constructed to simultaneously measure adjacent million neuron-sized fields of view so as to sample densely from most of the neurons in the mouse cortex, albeit still at coarse temporal resolution. More practically, approaches such as COSMOS that approximate cellular resolution but cover huge spatial extents may find increasing utility^23^. By combination of large field of view OEG methods with electrode arrays^159,160^, two-photon imaging^149^ or voltage imaging, many multiregion experiments that require different kinds of information from different brain areas are already feasible. Over the next decade, trade-offs among these rapidly advancing methods will likely become less significant as the field moves ever closer to the goal of comprehensively recording neural activity across the entire brain. But regardless of the ultimate method chosen, the question of how to best analyse multiregion data will remain a pressing concern (given these vast datasets), thus representing another area that has seen many innovations in the past decade^23,162^.

## Analysis techniques

Embedded in the choice of a data analysis method, and of each processing step, is a set of assumptions and biases for looking at the brain in a particular way. In this section, we present a taxonomy of existing neural analysis approaches that apply to cellular-scale datasets spanning multiple brain areas. Unique challenges and opportunities have arisen with the advent of multiregion cellular-scale data streams. Our intention is to articulate how particular analysis strategies are more appropriate for some kinds of questions about multiregion population coding than others. Importantly, selecting an analysis strategy implicitly restricts the hypotheses that can be tested, although this is often not explicitly acknowledged.

### Analysis strategies for multiregion data

Historically large-scale neural recording approaches include indirect haemodynamic methods such as functional MRI for imaging and bulk recording methods such as electroencephalography or field recording for electrophysiology. These approaches have permitted interrogation of brainwide circuits and systems — but at spatial and temporal resolution orders of magnitude coarser than for cells and spikes. As discussed earlier, recent advances have changed this landscape, presenting new approaches for relating the information contained in the spike trains of individual, genetically defined neurons to computations performed by brainwide networks.

Recent analyses of multiregion cortical datasets have revealed that many phenomena previously thought to be fairly restricted to specific brain regions are actually present across many areas^18,21–23^. Our capacity to understand these results is rapidly expanding now that we have access to multiregion data. For instance, the limited field of view of conventional two-photon imaging typically requires asynchronous sampling from distinct brain regions, and thus only trial-averaged responses can be compared between areas. However, such trial-averaged comparisons may bear little resemblance to true moment-to-moment correlations measured using simultaneous multiregion data^23^. While low spike-train correlations do not necessarily prove the existence of low levels of shared input^163,164^, such results do suggest that there is much for us to uncover regarding how the downstream actions of a particular neural circuit may yield different behavioural outputs, depending on the context^165,166^; new analytical approaches will be key for successfully performing large-scale single-trial analyses instead of pooling data across trials and animals.

**Fig. 2 |:**
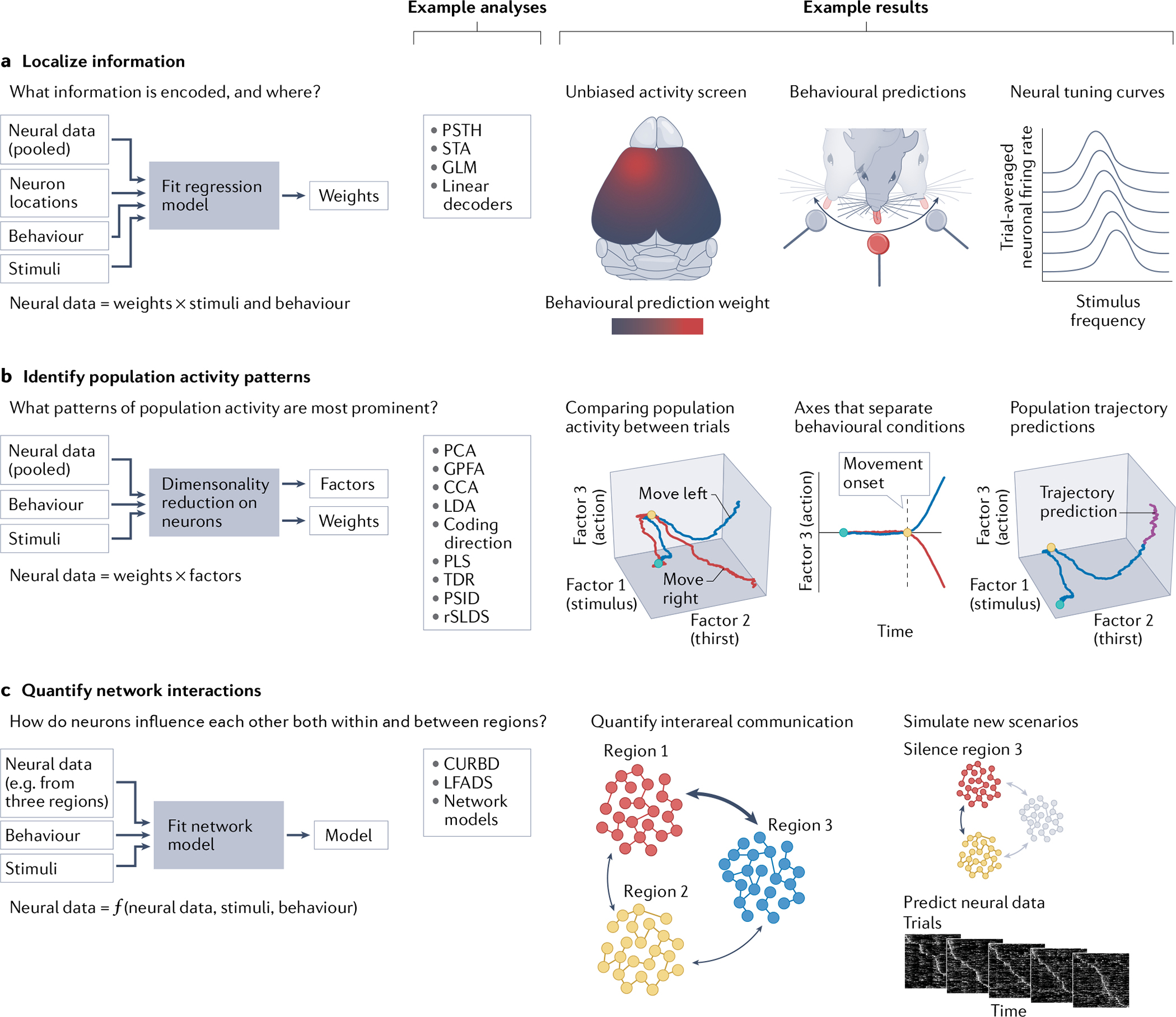
Approaches for analysing multiregion recording data. There are many approaches for analysing and interpreting multiregion neural data, each of which can address different types of questions. **a** | What information is being represented by neurons in a dataset? Questions of this nature can be studied using regression models that try to model neural data as a weighted sum of other variables, including information about animal behaviour, experimental stimuli, information about neuronal identity and position, and signals from other neurons. In the unbiased activity screen example (left), the weights obtained from a model might represent the brain areas that are most useful for predicting information about an animal’s behaviour during a task. In other cases (middle), the product of another set of regression weights and neural data may be used to make behavioural predictions (for example, to predict which waterspout the animal might lick to obtain a reward) or compute a neural tuning curve to show the average response of a neuron to a stimulus (right). The algorithm used to compute these weights is often some form of linear regression, such as a generalized linear model (GLM), and may rely on spike-triggered averaging (STA) to reveal the stimulus that a neuron maximally responds to or may rely on computing a trial-averaged response (often called a ‘peristimulus time histogram’ (PSTH)) to reveal the neural response to a specific stimulus. **b** | What is the regularity and prominence of different patterns of neural population activity? To study this question, dimensionality reduction techniques can be used to compress the activity of hundreds of neurons into a few prominent factors that can then be used to represent the joint activity of a neural population as a low-dimensional trajectory. These algorithms typically attempt to approximate a data matrix of neurons over time (*N* × *T*) as the product of a matrix of neuron weights over factors (*N* × *D*) and a matrix of factors over time (*D* × *T*), where the number of factors (*D*) is typically set to be some number less than *N* (often 2–5). This can be accomplished (in use of many algorithms) with different constraints on the features that must be present in the weights and factors (which are sometimes formulated in slightly different ways and may also incorporate information about behaviour, stimuli and how neural signals evolve over time). These methods, which are described in the main text, include principal component analysis (PCA), Gaussian process factor analysis (GPFA), canonical correlation analysis (CCA), linear discriminant analysis (LDA), coding direction analyses, partial least squares regression (PLS), targeted dimensionality reduction (TDR), preferential subspace identification (PSID) and recurrent switching linear dynamical systems (rSLDS). Projecting the neural data into a low-dimensional space defined by factors can be used to construct neural trajectories that can be visualized, quantified and used to compare the joint activity of a neural population across different trials and behavioural conditions (the green dot represents trial onset, the yellow dot represents movement onset and the red and blue lines represent schematic trial-averaged population activity as a mouse prepares to move right or left, respectively; left panel). Specific factors may be constructed to maximally separate population activity trajectories during different behavioural conditions (middle; format matches left panel). Many of these models can also be used to predict how a neural population trajectory might evolve in the future — in the absence of additional neural data (rightmost panel; general form of trajectory matches left panel). **c** | How do neurons interact with each other both within and between different brain areas? This can be studied by using algorithms such as latent factor analysis via dynamical systems (LFADS) and current-based decomposition (CURBD) to fit network models to datasets consisting of multiregion neural data, information about animal behaviour and sensory stimuli. The fitted network models can then be used to simulate how neural data might be generated by novel sets of stimuli or to generate unique behavioural outputs. Unlike with a real neural dataset, no element of these models is unobservable. Therefore, direct analysis of these models as a surrogate for the neural data of interest can be used, for example, to quantify the direction and strength of interareal communication between distinct brain regions (left). Communication strengths are indicated by the widths of the arrows between the areas. Use of these models also permits simulation of new experimental scenarios (for example, the behavioural and network-wide effects of silencing a set of neurons; right).

To analyse large neural datasets and attempt to answer these questions, at least three general approaches are relevant (FIG. 2). First are approaches for localizing information, largely based on computing correlations between recorded neurons and external covariates such as measured behavioural and stimulus features (FIG. 2a). This approach can be applied by fitting predictive models to test specific hypotheses, or by more exploratory analyses ranging from trial averaging based on behavioural structure to the study of neuronal firing across different temporal epochs of a dataset. Second are approaches for identifying population activity patterns — either by examining how neurons fire relative to one another or by examining how groups of neurons fire with respect to coincident behaviour (FIG. 2b). These algorithms enable visualization, description and modelling of the joint activity of groups of thousands of simultaneously recorded neurons. Third are approaches for quantifying network interactions that occur both within and between different brain areas (FIG. 2 c). One increasingly popular approach here is to use new modelling techniques that can be fitted to large neural datasets. The resulting models match many features of the neural data but are more amenable to analysis and understanding. Therefore, these models can be rapidly analysed and experimented on in silico before the predictions are tested in new biological experiments. We discuss each analytical approach in turn.

### Localize information

A common goal in many of these analyses, whether for large or small datasets, is to relate information about sensory stimuli or behavioural output to recorded neural activity (FIG. 2a). This can be accomplished through a variety of means, but a key feature of many analysis methods is that they are dependent on correlations within the data. The simplest and perhaps most common form of correlational analysis is the notion of a trial-averaged response. This idea dates back more than a century to the original notions of neuronal tuning curves and receptive fields^167,168^. Beyond this basic idea of plotting average neural responses against stimulus properties^169^, the more general idea of computing a stimulus-triggered average firing rate is commonly used and is often referred to as the ‘peristimulus time histogram’ or developed into various elaborated forms^170,171^. These kinds of analyses do not historically treat neural population data any differently than a set of individual neurons. Tuning curves can be simply computed independently for each neuron, or by averaging over multiple neurons. But with new multiregion datasets, understanding unaveraged single-trial responses is of particular interest. Fortunately, a host of modern correlation-based approaches are designed to work in this newer setting.

Many analyses along these lines can be dichotomized into those that attempt to predict neural activity from stimulus and behavioural features (often called ‘encoding models’) and models which predict stimuli and behaviour from neurons (‘decoding models’)^18,21,22,34^. Traditionally, encoding models attempt to predict the response of a single neuron at a time with different combinations of task features — and are fit using regression algorithms. In contrast, decoding models more obviously lend themselves to the analysis of a whole neuronal population, because a simple regression scheme could be used to predict a single task feature from many simultaneously recorded neurons (potentially spanning multiple areas). But over the past decade multiple techniques have been developed to generalize encoding models to neuronal populations. Fitting encoding regression models to whole populations at once, rather than treating neurons independently, has been found to generate better predictive performance^172^. In a similar way, the performance of encoding models can be improved by incorporating known information about the structure of a neural circuit and the statistics of spike trains (for example, by using Poisson–generalized linear models (GLM))^172,173^. Variants of this approach can be specifically constructed to account for interregional connectivity and unknown time lags between neurons in the case of multiregion data^174–176^.

This process of building, fitting and analysing encoding models can reveal much about the processes that might generate observed patterns of neural activity — and has become increasingly common as available software packages have made such models easier to implement^177–179^. In the past, these methods were most frequently applied to trial-averaged data, or even measurements pooled between recording sessions or experimental animals. More recently, new recording methods have enabled the acquisition of sufficiently large datasets to perform these analyses on simultaneously measured neurons from single sessions. As a whole, this increase in scale is significant because it enables experimentalists to perform unbiased activity screens, by analogy with unbiased genetic screens that have been so useful in other fields of biology, here to determine which brain regions might be functionally implicated in a behaviour of interest^180^.

Regardless of the specific analysis approach used, working with large multiregion datasets presents new challenges. For example, if one were to rely on hundreds of pairwise statistical tests to assess whether significant differences might exist between the firing patterns of neurons, it would be necessary to account for the chance of false positive comparisons by using a false discovery rate correction. In a similar way, when one is working with hundreds of slow, time-varying neural signals, a key concern to keep in mind is that of ‘nonsense correlations’^181^. As many correlation metrics that are commonly used assume each time point is independent of all others (which is obviously false for filtered data), it is often possible to observe strong correlations between time-varying neural signals and even unrelated time-varying variables (for example, the stock market or the price of a cryptocurrency^182^). Without appropriate control analyses, these kinds of correlations may be erroneously judged as significant. Simple controls where one signal is randomly shuffled to generate a comparative ‘null distribution’ are often insufficient if there is clear time-varying structure imposed in a neural dataset by a stimulus or stereotyped behavioural response. Stronger controls that preserve long-term structure, such as shift permutation or trial and session shuffling, can help mollify such concerns^181^. The observation that ongoing movements explain a large fraction of the variance in neural activity across the brain further highlights the need for careful behavioural task design — and analysis strategy choice^183^. In general, both experiments and subsequent analyses should be designed with appropriate controls to ensure that reported results based on correlations do not simply occur due to chance.

### Identify population activity patterns

As datasets increased in size over the past decade, new approaches for describing the joint activity of thousands of neurons were developed. In particular, population firing-rate trajectories became increasingly common tools for modelling the joint firing of whole neural populations^184^. This approach is quite practical, as it permits compression of the joint neural activity from even hundreds of neurons to something that can be visualized on a 3D plot (FIG. 2b, left). The most common means of projecting high-dimensional neural data into a low-dimensional space is principal component analysis (PCA) — which performs linear decomposition on the covariance structure between neurons to define a set of orthogonal axes (often called a ‘latent-variable space’, as the variable representing each axis is inferred, rather than directly observed) where each explains as much variance as possible in the data. This approach is highly effective in neural systems. Across many brain areas and behavioural tasks, most of the variance in the firing of hundreds of neurons can be explained using far fewer dimensions than the number of neurons^18,21,185–187^. However, there are important limitations to this approach.

First, the process of estimating a smooth ‘neural trajectory’ that represents the evolution of population activity over time requires more specialized methods than standard PCA. One such approach is Gaussian process factor analysis (GPFA) — an algorithm that simultaneously identifies basis vectors and defines a smooth neural trajectory^188^. In a similar way, methods based on canonical correlation analysis can identify shared neural dimensions between different datasets (which might not share any neurons), such that neural trajectories measured in a brain area could be aligned among different datasets and permit changes in neural dynamics to be tracked for many months or even years^189^ or to compare trajectories between different areas^174^. Along these lines, the recurrent switching linear dynamical systems model attempts to decompose neural population trajectories into segments that can be approximated by models with linear dynamics^190^. This approach has been found to identify states of neural activity that correlate well with manually labelled behavioural states not used to train the model^191^.

Second, performing dimensionality reduction and then quantification on a resultant trajectory makes two strong assumptions about the data. First, it assumes that the signal of interest is low-dimensional (for example, that the joint activity of 300 neurons can be summarized by three time-varying signals). Second, it assumes that the specific dynamics of the neural trajectory capture relevant features of the data under study^192^. This second assumption depends on the strong hypothesis that smooth firing rate dynamics, rather than features of precise spike timing, contain the neuronal population codes of interest. We know that this assumption is often at least partially wrong, as single-neuron spike timing codes that have been observed in various experimental settings are eroded by most common methods for smoothing spike trains into firing rates (which often assume Poisson-like spiking statistics that do not necessarily match the data)^193,194^. Nevertheless, dynamical models that make these assumptions constitute an exciting area of computational neuroscience research because there is much emerging evidence that the evolution of these neural trajectories over time may indeed describe certain neural computations^195^.

However, interesting structure apparent in neural trajectories based on just a few dimensions need not remain in the full high-dimensional dataset, and other unappreciated features may exist in the data beyond a single trajectory^184^. One solution to this problem is to use complementary analyses that operate on many more dimensions than were visualized, or on the full-dimensional neural space^128,162,185,196^. Empirical methods also exist for testing whether novel claims about population codes (for example, based on fitting latent-variable models) are potentially explainable by known features of single-cell response properties^197^.

So what is the relevant dimensionality of the neural activity in a given brain region? This parameter is likely to depend on the neural structure under study (for example, sensory versus motor) and the complexity of the behaviour^186,192^, as well as technical details such as the temporal resolution of the data acquired (for example, resampled second-long time bins versus short single-spike time bins). To approach these questions, other dimensionality reduction schemes (besides PCA) may be of use, which quantify the differences in neural activity between distinct experimental conditions — regardless of whether they explain most of the variance in the data (which is the goal of PCA). For example, neurons across the dorsal cortex encode motor-related information such as the current location of a reward, but this explains only a small fraction of the total variance in the data^23^. In this case, PCA is inappropriate, and using it to draw low-dimensional neural trajectories may not yield any obvious differences in population activity between different experimental conditions.

What alternatives to PCA are available? One approach, linear discriminant analysis, seeks to find an axis (the ‘linear discriminant’) that best separates data points on the basis of some covariate, such as lick direction. This approach works for arbitrary numbers of conditions, but if there are only two conditions to separate in the data (for example, lick left or lick right), the linear discriminant can be approximated by simply computing the vector difference between mean activity under the two conditions. This is sometimes referred to as the ‘coding direction’^198^. Similarly, an algorithm called ‘partial least squares regression’ is a common approach^23,199,200^ for jointly achieving two goals: (1) finding a low-dimensional representation of the data that explains much of the variance in the data and (2) using the data in that low-dimensional space to solve a regression problem (that is, to separate trajectories from different experimental conditions). Related algorithms that attempt to jointly find a hidden or ‘latent’ state space that explains variance in the data but also separates the data along experiment-defined conditions include demixed PCA, tensor component analysis and targeted dimensionality reduction^201–203^. A recent method, preferential subspace identification (PSID), has developed a dynamical model for identifying low-dimensional neural trajectories that also incorporate behavioural information, using measurements of the animal’s behavioural dynamics to aid in the identification of task-relevant neural dynamics^204^. Interestingly, a nonlinear variant of PSID (which relies upon recurrent neural networks (RNNs) used in deep learning models) performs similarly to the linear variant of the algorithm in mapping cortical activity into a latent space. Only behavioral decoding is significantly improved by the use of a nonlinear model — suggesting that cortical dynamics may be readily explainable by linear dynamics, but that transformations from cortical activity to behavior may be particularly nonlinear^205^.

More work is required to further generalize these approaches to explicitly multiregion data. One key issue is that many current approaches treat neurons identically, without regard for known differences between neurons residing in different brain areas or those with different gene expression profiles. Alternatively, the neural activity within each region is reduced to just a single signal (or a small number of signals per unit area). This use of a single time-varying scalar for coupling areas makes it easier to know that interregional interactions may be occurring — and could enhance the experimental use of closed-loop interventions in health and disease^206^ but comes at the expense of knowing what information might actually be transmitted^207^. But it is likely that the mechanisms that govern information flow between areas in the cortex are not the same everywhere. Thus, multiregion models that treat the neural activity from different brain regions distinctly are necessary — especially since we know there are clear anatomical and physiological distinctions between brain regions. For example, the motor cortex and the spinal cord are coupled via many ascending and descending neural pathways, but it seems unlikely that either region forms the majority of the inputs to the other under any behavioural circumstance; therefore, using only a single set of latent factors to represent a dataset comprising recordings from both areas would make little sense. Developing richer models that can incorporate this kind of information will be critical for building robust brain-machine interfaces and neural decoding algorithms that achieve high performance in complex, real-world scenarios — in addition to guiding us towards a better understanding of the brain.

### Quantify network interactions

Beyond quantitatively describing population codes within distinct brain areas, a second-order set of questions seeks to understand how brain areas communicate with each other. New analyses will be important for identifying the mechanisms of interregional communication, and for testing several major hypotheses regarding corticocortical communication^208^. Three main mechanisms have been proposed. First, correlations between the spike trains of neurons in different areas seem to facilitate information transfer^209,210^. Second, coherent oscillations, particularly in the gamma band, may enhance information transmission by cortical neurons^211,212^. Third, interareal communication (both between cortical areas and in cortical–subcortical pathways) can occur within a ‘communication subspace’ such that projection neurons usually fire in a pattern whereby their net effect on a downstream area cancels out (that is, firing in the null space of the postsynaptic area) — except when they are actively broadcasting information^213,214^.

Each of these mechanisms, in addition to the operation of potential ‘gate’ neurons in pathways beyond the cortex (for example, ‘omnipause’ neurons in the brainstem that gate descending inputs during eye movements), likely plays a role in different behavioural circumstances^215^, and may now be accessible using multiregion cellular-scale methods. This question of how areas communicate is intertwined with the question of what information is communicated. However, it is still very much an open question whether or how upstream areas ‘command’ downstream areas^216^ or whether some interareal connections may influence other areas in a subtler manner, for example, via gain regulation^217^.

Methods from network analysis and topology may be applied to multiregion neural datasets to address these questions. Either simple correlation-based metrics or other, related metrics such as Granger causality, or information theoretic quantities, can be used to quantify directional dependencies between individual neurons or areas, and then network models can be defined using either neurons or brain areas as nodes and with the chosen metric defining functional connections between them. These network models may be useful to develop schemes for controlling the brain, or for better understanding its function^218^. Indeed, a whole host of network models at different levels of complexity may be applied for better understanding different aspects of interregional communication^219^.

However, multiregion neural datasets present a particular problem that hinders many kinds of network analyses — the fact that mammalian multiregion recording techniques afford only the ability to incompletely sample from a subset of neurons in a subset of brain areas. One emerging approach is to use these incomplete multiregion neural datasets (which have not measured every relevant activity parameter) for training recurrent neural network (RNN) models that can be then perturbed and analysed in silico (FIG. 2c). While this core idea of building detailed computational simulations of neural circuits can be taken to highly detailed and biophysically realistic levels^220^, these RNN-based methods usually seek to model features of neural and behavioural responses by using modern deep learning methods, rather than by creating explicit models of biological neurons^221–231^. For example, in the widely used latent factors analysis via dynamical systems (LFADS) framework, individual artificial units do not represent individual biological neurons^221–224^. Rather, as Sylwestrak and colleagues recently demonstrated^224^, LFADS can be used to directly model the underlying dynamical systems corresponding to distinct biological neural populations. Another approach (current-based decomposition) maintains a one-to-one correspondence between biological neurons and artificial units during model fitting, but this procedure is used to generate a 1D time-varying interaction signal between different brain areas (regardless of the number of neurons fitted per area)^230^. Even in the case of incomplete sampling from the brain, these approaches fit available multiregion neural data to analytically tractable ‘surrogate’ models that can be used to generate testable hypotheses for future experiments.

This approach of building a surrogate model that can generate known neural dynamics is also appealing because RNN models have become increasingly amenable to detailed analysis. For example, specific low-dimensional dynamical motifs can be reliably identified in trained RNN models as learned solutions to many common language processing and neuroscience-inspired tasks^225–228^. However, some of the analytical tools used to find these motifs are difficult to apply to real neural datasets. For example, dynamical fixed points and basins of attraction may be hard to identify in cortical areas because the area-specific recurrent dynamics are usually happening in the presence of strong external input from other, often unobserved, brain areas or sensory systems^229^. But surrogate neural models, such as current-based decomposition^230^, that explicitly model multiple brain areas (which need not be at cellular resolution) may offer a path forward here: a multiregion RNN model can be fit to a large neural dataset and then analysed in situations wherein external inputs to a brain area of interest are disabled, or perturbed in other ways^223,231^. Optogenetic manipulations could then be used to experimentally validate a tractable set of predictions made by the model.

At a more abstract level, RNN models that generate behavioural data can be used to test different neural analysis strategies. A combined experimental and computational article that validated this analysis paradigm emerged from the study of larval zebrafish, where single-cell neural activity can be measured almost comprehensively from the entire brain and spinal cord^45^. By the fitting of an RNN to activity measurements from most neurons in the zebrafish brain, distinct changes in the coupling strength between the habenula and raphe nucleus could be seen as fish entered a depression-like state, passively rather than actively coping with a stressor, in the process clearly identifying a circuit previously hypothesized to be involved in depression and passive coping. Importantly, because this approach processes data from all regions across the brain in an identical way, this brainwide analysis was not biased towards any particular answer. At a less comprehensive level, multiregion RNNs have also been used to reproduce interregional network dynamics within both the mammalian cortex and subcortical areas^198,232–234^. Finally, causal tests will be crucial in validating these models. Recent work experimentally perturbed information flow between two areas of the visual cortex (V1 and LM) by inhibiting activity in one area, at different time lags relative to a visual stimulus^235^; influence between the areas was observed to vary over time — much analogous to how recently developed models have been used to estimate time-varying ‘currents’, or lagged latent variables, that link brain areas^230,236^.

Moving forward, a key question is how complete the neural population recordings must be to build a model of the type described here that can accurately recapitulate population dynamics. Explicit incorporation of neuronal cell-type information to delineate subpopulations may be useful in this way. Of similar importance is a clear means of identifying the number of brain areas present in a dataset. Changing the definition of a brain region in this context will certainly influence any measures of interregional communication^219^. As discussed earlier, using a common reference atlas framework to delineate gross anatomical areas is an important first step. But when one is building an RNN model, is it best to try to learn regional groupings between neurons from the data themselves? Or must granular anatomical labels (for example, cortical layer within an area instead of just areal identity) be applied^230,231^? To some extent, the answer to these questions will depend on the biological questions of interest, the regions being studied and the behavioural state of the animal. However, it is exciting to consider the idea that as this framework increases in sophistication, single models may be able to accurately model neural activity during complex behaviours, as well as during a variety of perturbations to the relevant neural circuitry (for example, activation or silencing of different brain areas) or behavioural setting (for example, across different environments). If this potential can be realized, studying RNN models as a surrogate for new experimental data will be a tremendously powerful tool for systems neuroscience.

## Recent findings and future directions

**Fig. 3 |:**
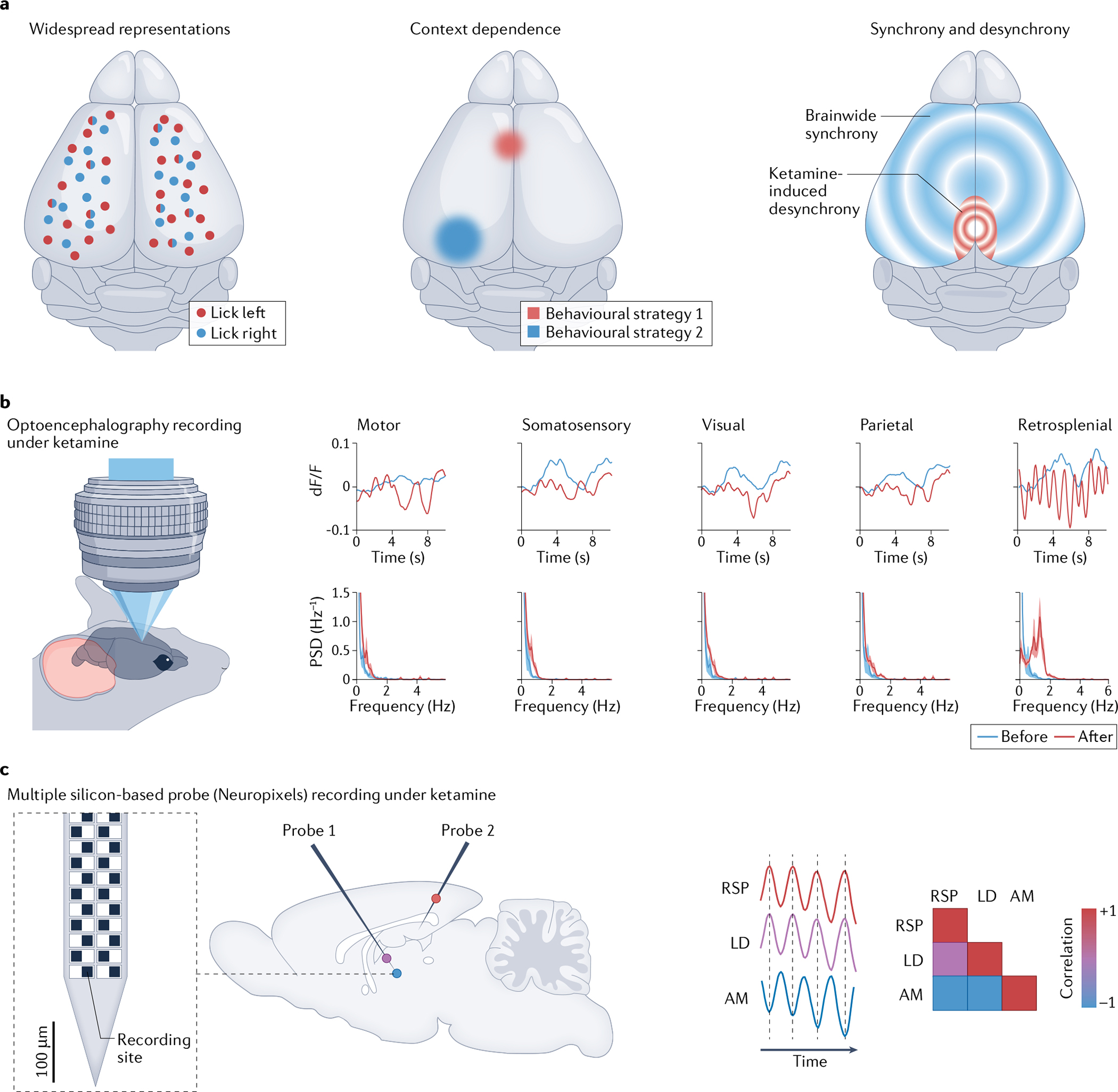
New perspectives arising from multiregion recording. **a** | Example insights thus far into brainwide activity. Widespread nature of state and stimulus representations, here shown as widespread encoding of different actions (left; as in^23^). Context dependence of interregional dynamics, here shown as different patterns of regional dynamics depending on the behavioural strategy (such as an active or a passive detection strategy, as in^243^; middle). Roles of synchrony, here shown as desynchronized rhythmic activity between the retrosplenial cortex (red oscillations) that are decoupled from activity in other cortical regions (blue oscillations) that was observed to be elicited by ketamine, a dissociative drug (as in^180^; right). **b** | An unbiased activity screen using optoencephalography widefield imaging reveals a ketamine-elicited (50 mg kg^−1^) rhythm localized to one cortical region, yielding desynchrony between the retrosplenial cortex and other cortical regions. Ketamine’s effect is an important example of the value of multiregion imaging since the uniqueness of the effect seen in the retrosplenial cortex would not have been otherwise appreciated, and also could not have been predicted. This effect is evident in the top row as a sinusoidal pattern of activity in the retrosplenial cortex after infusion (red trace) that is not correlated with the post-infusion activity of other regions. In the bottom row, this is exhibited as a peak at 1 Hz in the spectral power of the activity after infusion (red trace). d*F/F* is the baseline-corrected change in fluorescence, blue traces are for before ketamine infusion and red traces are for 10 min after ketamine infusion. **c** | Recordings with multiple Neuropixels silicon-based probe electrodes further reveal ketamine-elicited correlation between the retrosplenial cortex (RSP) and the laterodorsal thalamus (LD), and inverse correlation between the retrosplenial cortex and the anteromedial thalamus (AM). The inset illustrates a Neuropixels silicon-based probe (version 1.0) with a dense arrangement of electrodes that enables recording from many individual neurons across multiple regions of the brain (indicated by colours that correspond to the data shown on the right). See REF^180^ for further information on recording and analysis details. PSD, power spectral density. Panel **b** reprinted from REF. ^180^, Springer Nature Limited. Panel **c** adapted from REF. ^180^ and REF. ^151^, Springer Nature Limited.

Initial studies applying multiregion recording methods in different behavioural contexts have begun to demonstrate the types of findings that can result from a broad, brainwide perspective. At least three major themes have emerged (FIG. 3). First, many behavioural features and stimuli have widespread neuronal population representations, and are decodable from neuronal dynamics in seemingly surprising locations across the brain. Second, the location and content of multiregion neural representations and dynamics depend on behavioural context. Third, specific interregional patterns of synchrony and asynchrony appear to be important features of behaviourally relevant neural dynamics.

### Widespread representations

One simple and powerful advantage of multiregion recording lies in surveying activity across many regions, in an unbiased way — thus including areas not expected to be particularly involved in a given behaviour. As a consequence of applying this approach, many recent studies have revealed that neural representations for various behavioural features are not confined to specific individual brain regions. For example, ongoing motor behaviour is represented not only in anterior motor areas — as expected — but also in posterior areas such as the primary visual cortex^21,22,237^. During a visual task, neurons in nearly all of 42 regions electrophysiologically recorded across the brain were observed to respond non-specifically when mice initiate an action^34^. Furthermore, even specific history-guided motor plans are encoded widely across the cortex^23^. Finally, sensory evidence appears to modulate activity in the secondary motor cortex in the absence of movement^238^. Whether these widespread signals subserve learning, context setting, distributed computation or even no behaviourally relevant purpose at all remains an important question well suited for future causal investigation.

Another important takeaway message from recent analyses of multiregion data is that neurons with similar trial-averaged activity patterns often display very different single-trial combinations of cognitive and movement variables^21^. For example, in one recent analysis of trial-averaged cortex-wide imaging data, there was no clear dependence of correlation strength over space — that is, pairs of neurons at near and far distances had high correlations. In contrast, single-trial correlations computed on the same dataset exhibited more localized structure^23^. Similarly, in a different experimental setting, spike-triggered maps (which are inherently trial averaged) from simultaneous electrophysiology and OEG displayed widespread cortical activity motifs related to the activity of individual thalamic or cortical neurons^239^. Thus, population-level signatures of behaviour are not only widespread; these signatures also manifest themselves differently on analyses of single-trial versus trial-averaged neural data.

What causal role do these widespread representations of behaviour play? Optogenetic interventions have the potential to provide important insight. As with multiregion recording, optogenetic manipulations have progressed to ever wider fields of view^128^ — even to cortex-wide scales^23^. Importantly, though, since brainwide activity patterns can arise from activity in localized populations of neurons (for example, sensory neurons, or neuromodulatory neurons that correlate with brain states such as arousal^240^), investigators can likewise readily generate naturalistic brainwide patterns of activity with even focal optogenetic interventions (if properly targeted). An example is a study using Neuropixels paired with optogenetics in which focal stimulation of input to the neurons of the subfornical organ with a single deep fibre optic triggered brainwide naturalistic internal representations of thirst, and of seeking water when thirsty^18^. These experiments illustrate how optogenetic interventions operate in ways fundamentally analogous to gain-of-function or loss-of-function genetic interventions (for example, gene knock-in/knockout, RNAi/short hairpin RNA and CRISPR–Cas genome editing) in other realms of biology^241^, wherein precise highly local perturbations provide insight into the global causal underpinnings of complex system function.

### Context dependence

Another key benefit of multiregion investigation is the enhanced ability to compare neural dynamics within different contexts. These contexts can include task difficulty, sensing strategy and behavioural state^242^. By simultaneously measuring activity across the brain, one can survey the context-dependent involvement and interactions of many regions and ensure that regional differences are not due to uncontrolled differences in context or behaviour that might occur with asynchronous recordings. Moreover, by recording joint activity across behavioural conditions, one can disentangle potentially complex behavioural variables that confound interpretation of population neural activity.

A number of studies have discovered patterns of multiregion activity that distinguish scenarios with similar stimuli or actions but differing higher-level context. For example, the difficulty of a task can alter how identical stimuli are processed, with widespread multiregion activity ramps and decreased correlation across the cortex during a more complicated evidence-accumulation task versus a simpler explicit visual response task^233^. Moreover, use of optogenetic inactivation to silence activity in single regions across the dorsal cortex influenced performance on the evidence

accumulation task. However, performance on the simpler task only depended on activity in a few visual cortical regions. The representation of a stimulus can also change depending on the strategy used by the animal for sensing the stimulus, as in one example where the locus of short-term memory encoding in the dorsal cortex changed depending on whether the mouse used an active or a passive whisking strategy to identify a texture, and targeted optogenetic inactivation could even induce the mouse to use a different strategy^243^. The temporal sequence of stimulus presentation can also impact multiregion neural representation, as during a delayed non-match to sample task wherein the secondary somatosensory cortex was sensitive to whether the second stimulus matched the first stimulus and appeared to relay recalled information to primary somatosensory regions^141^. Last, the degree of agency that an animal has over a stimulus can influence multiregion activity. Using a multiregion brain–machine interface, one study found that when the position of a cursor was controllable, higher visual areas were more active, cursor position was more decodable from population neural activity and units exhibited increased correlation with cortex-wide activity^244^.

The behavioural state of an animal can also influence multiregion activity. For example, when an animal locomotes, units in the primary visual cortex become more strongly coupled to motor and local visual cortical regions, whereas retrosplenial units become less locally coupled^159^. Task engagement can also globally influence cortical activity, eliciting desynchronization and persistently decreased low-frequency (3–6-Hz) activity^245^. Finally, motivational state, such as whether a mouse is thirsty or sated, impacts global activity patterns, leading to a brainwide ‘initial condition’ that influences the transformation of sensory input into behavioural output^18^.

### Synchrony and desynchrony

Perhaps one of the most valuable aspects of simultaneous multiregion recording is the capability to observe the details of correlated activity across the brain. Recording in two regions at the same time, such as the medial prefrontal cortex and hippocampus^246–248^, medial prefrontal cortex and ventral striatum^249^, frontal and visual areas^34^, or secondary motor cortex and posterior parietal cortex^250^ has already given rise to many synchrony-related insights, including into neuropsychiatric symptoms such as anhedonia^249^; indeed, synchrony and desynchrony have long been hypothesized to be important in neurobehaviourally important conditions such as schizophrenia, autism, depression and dissociative states. The advances now making it feasible to record from many more than two regions (for example, recordings with six simultaneously deployed Neuropixels probes revealing hierarchical structure in multiregion functional connectivity at the cellular level^33^, or widefield imaging^251^) promise an expansion of this perspective, both for basic science understanding and for insight into neuropsychiatric disorders. Further evidence for altered functional connectivity has been observed with multiregion recording in depression-related states, such as during recording from seven regions in a mouse model of stress response, which yielded multiregion activity factors that could serve as signatures for discriminating behavioural conditions^252^. Similarly, recording from five regions in a model of autism spectrum disorder yielded the discovery of diminished social stimulus-induced increases in coherence between the cingulate cortex, thalamus and nucleus accumbens^253^. Additionally, multiregion recording led to the discovery of a key role for desynchronized dynamics in the clinically important state of dissociation, whereby administration of dissociative drugs such as ketamine elicited a 1–3-Hz oscillation localized to the retrosplenial cortex (but not other dorsal cortical regions), a brainwide disappearance of most correlations with the retrosplenial cortex and an uncoupling of activity between laterodorsal and anteromedial thalamic regions^180^. Importantly, the mere presence of a slow oscillation in the retrosplenial cortex was not the distinguishing factor, but rather the spatial restriction of the oscillation and its desynchronization from other cortical regions was the distinguishing factor. Indeed, these multiregion recording observations were critical for informing the design of causal optogenetic and gene knockout experiments that pinpointed the role of the retrosplenial oscillation in dissociation-like behaviour, guiding analysis of multiregion intracranial electrophysiological recordings in the dissociating human brain and the discovery of similar oscillations in the homologous human retrosplenial and deep posteromedial cortical regions.

Looking forward, there are many opportunities for investigating the roles of synchrony in disease states. For example, since altered interregional brainwide communication has been long hypothesized to be relevant to the symptoms of schizophrenia and other psychotic states, it will be interesting to test whether multiregion relationships are altered in preclinical or clinical states with perceptual alterations, including during administration of psychosis-inducing pharmacological agents.

## Conclusion

The mammalian brain is a complex system composed of many interdependent parts. In such systems, macroscopic properties emerge from properties and interactions of the individual parts of the system, and the state of each part depends on the state of the others. Neuroscientists may now draw upon new methods to investigate how dynamics of the whole brain and the behaviour of the animal depend on interactions among elemental parts. To advance this goal, here we suggest that it will be crucial to see the parts and the whole at the same time — in particular, by measuring cellular activity in multiple brain regions at once. This integrative approach, encompassing optical, electrophysiological and computational innovations, enables new types of observations, such as measurements of distributed population codes and of interregional synchrony, which are inaccessible to methods that probe one region or cell at a time. Especially when paired with optogenetic control^241^, multiregion recording provides a vital source of information on naturally occurring brainwide activity patterns that can be screened for in an unbiased fashion^180^, and then tested for causal significance in physiology and behaviour. Ultimately, by using the experimental and computational approaches discussed here, we have the opportunity to see both the forest and the trees of the brain — emergent brain-spanning states and their constituent cellular dynamics — at the same time.

## Figures and Tables

**Table 1|:** Multiregion recording techniques present distinct trade-offs

Method	Acquisition speed	Spatial resolution	Spatial coverage	Cost and complexity	Genetic specificity	Freely moving	Example refs.
Multiple Neuropixels probes	More than kilohertz	Single cell	Select regions throughout the brain, along multiple linear trajectories	Specialized mount, multiple fragile probes (each ~$1,000), acquisition hardware (<$50,000), relatively simple burr-hole craniotomy surgery	Possible with optotagging	Up to two probes currently, probably more possible	22,180,224
Two-photon mesoscope	<10 Hz	Single cell	A few adjacent cortical regions (~5-mm diameter)	Custom large microscope objective, high speed optomechanics and detectors, lasers, large optical table, more than hundreds of thousands of dollars, commercial system available, large glass window surgery	Yes, with viral or transgenic reporter expression	Not currently	138,139,142,143
Multi-objective two photon imaging	>10 Hz	Single cell	A few select regions in the cortex, or accessible with a GRIN lens	Custom optics and optomechanics, lasers, large optical table, more than hundreds of thousands of dollars, multiple glass window or GRIN-lens surgery	Yes, with viral or transgenic reporter expression	Not currently	136
COSMOS	>10 Hz	A few cells	Entire dorsal cortical surface (~10-mm diameter)	Large-sensor sCMOS camera, lenslet array (<$1,000), minimal alignment, small spatial footprint, total <$50,000, large glass window surgery	Yes, with viral or transgenic reporter expression	Not currently	23
Single Neuropixels probe	More than kilohertz	Single cell	Select regions throughout the brain, along a single linear trajectory	Fragile probe (each ~$1,000), acquisition hardware (<$50,000), relatively simple burr-hole craniotomy surgery	Possible with optotagging	Yes, including probe reusability	54,56,151,224
Optoencephalography (widefield imaging)	>10 Hz	Bulk activity of many cells	Entire dorsal cortical surface (~10-mm diameter)	sCMOS camera, minimal alignment, small spatial footprint of hardware system, total <$50,000, simple surgery	Yes, with viral or transgenic reporter expression	Yes	6,16,48,58,83
Multifibre photometry	>10 Hz	Bulk activity of many cells	Select regions throughout a brain	sCMOS camera, fibre bundle, commercial systems (<$20,000), multiple fibre implant surgery	Yes, with viral or transgenic reporter expression	Yes	67,111,113
Photoacoustic imaging	>10 Hz	Bulk activity of many cells	Whole brain	High-power pulsed laser, custom ultrasound transducer array, non-invasive preparation with intact skin and skull (~$350,000 total)	Yes, with viral or transgenic reporter expression	Not currently	133

COSMOS, cortical observation by synchronous multifocal optical sampling; GRIN, gradient index; sCMOS, scientific CMOS.

## Box 1 | Processing multiregion neural data

A new generation of mostly automated data-processing pipelines has become available to efficiently analyse multiregion neural datasets of increasing size.

### Extracellular electrophysiology and spike sorting

Starting with raw multielectrode voltage data, spike waveform-specific bandpass filters are applied before a spike detection algorithm (often just a threshold) is run to identify candidate action potential times. This leaves the final step of ‘spike sorting’: the process of taking each extracted spike and its waveform shape and sorting them into distinct lists of spikes (called ‘units’) that share similar properties. The resulting units are the starting point for most subsequent analyses. There are now many competing approaches for semi-automated spike sorting^56,254–259^, but because it is a difficult problem, no single algorithm is appropriate for every experimental scenario^257^. New pipelines that include many alternative algorithms make it easier to compare many approaches on a single dataset^254^.

### Two-photon Ca^2^+ imaging and source extraction

Many algorithms^114,260–262^ now automatically identify the location and shape of each neuron present in a two-photon fluorescence video. Some pipelines also perform a final step, which is to perform ‘spike inference’ and estimate a discrete-time firing rate histogram for each neuron so as to recover information that might have been smoothed out by the slow temporal kinetics of the Ca^2^+ sensor^263–265^. A recent study acquired data wherein two-photon Ca^2^+ imaging was performed before electron microscopy provided a ‘gold standard’ for quantitatively evaluating spatial source extraction algorithm performance^266^. Benchmarks quantifying spike inference algorithm performance are also available^267,268^, as are other packages for generating realistic simulated data that can be used for comparing different analysis approaches^269^.

### One-photon Ca^2^+ imaging and source extraction

Single-photon Ca^2^+ fluorescence videos can vary dramatically — depending on whether they are obtained from a small field of view endoscope implanted deep in the brain or from a widefield microscope. In either case, the signals observed in a given pixel are likely to have arisen from many neurons stacked on top of each other in space. Recent work has involved the development of statistical inference tools^270–272^ to separate in-focus neural signals from deeper out-of-focus noise — and to extract a single denoised trace for each detected neuronal source. Manual validation of components extracted with any processing approach here is especially critical, especially if motion artefacts are significant.

## Box 2 | Which multiregion recording technique should you use?

### Spatial resolution: many multi-unit signals versus fewer unambiguous single units

Cellular resolution data are critical for questions pertaining to single-neuron tuning, and for relating physiology to cell-type identity. But when the goal is to decode information rapidly, to compare activity between regions or to study population codes under different behavioural circumstances, multi-unit spiking data or multineuron imaging techniques such as cortical observation by synchronous multifocal optical sampling may offer many more signals than cellular resolution approaches — and still yield suitable data for many population analyses^23,162^.

### Spatial coverage: dense, slow data versus sparse, fast data

Silicon electrode arrays such as Neuropixels measure activity from a sparse subset of neurons near the probe, with straightforward access to subcortical regions in the mouse. In contrast, a two-photon microscope can see vastly more neurons per unit area. But this increased spatial coverage comes at the expense of temporal resolution: scanning a laser across an entire plane or volume takes many milliseconds, whereas each electrode on an array can be recorded at kilohertz data rates. More generally, scanning a laser over a volume, while remaining at each pixel long enough to measure a useful signal from any neurons that may be present there, enforces a trade-off among temporal resolution, coverage density and area sampled.

### Temporal resolution: many neurons at low speeds versus few neurons at high speeds

While Ca^2^+ imaging permits dense access to cellular resolution activity measurements from genetically defined populations, this comes at a cost. Most existing Ca^2^+ sensors are unable to reliably measure single action potentials in single trials^114–116^, independent of the data acquisition rate. Ca^2^+ sensors report a noisy proxy of neuronal firing passed through a nonlinear filter^114,267,268^. Despite advances in voltage imaging, electrophysiology remains the ‘gold standard’ for recording with single-spike resolution.

### Optogenetic compatibility: writing neural activity without compromising read capability

Shining a light near an electrode array can generate stimulus-locked electrical artefacts via photovoltaic, photoelectrochemical or electromagnetic effects^273,274^; however, recently designed electrodes appear fairly resilient to this issue^151,274^, and recently developed ultrasensitive opsins permit the use of reduced light intensities for photostimulation^128,275,276^. Similarly, new spectral variants of Ca^2^+ indicators, such as XCaMPs and sRGECO^73,277^, have increased compatibility with optogenetic tools — particularly in conjunction with red-shifted channelrhodopsins^128^.

## References

[1] The Event Horizon Telescope Collaboration. First M87 event horizon telescope results. I. The shadow of the supermassive black hole. Astrophys. J. Lett. 875, L1 (2019).

[2] Galilei G Sidereus Nuncius (Univ. Chicago Press, 1610).

[3] Adrian ED The Basis of Sensation (WW Norton & Co, 1928).

[4] Brock LG, Coombs JS & Eccles JC The recording of potentials from motoneurones with an intracellular electrode. J. Physiol. 117, 431–460 (1952). 12991232 10.1113/jphysiol.1952.sp004759 PMC1392415

[5] Woodbury JW & Patton HD in Cold Spring Harbor Symposia on Quantitative Biology vol. 17, 185–188 (Cold Spring Harbor Laboratory Press, 1952). 13049165 10.1101/sqb.1952.017.01.018

[6] Ren C & Komiyama T Characterizing cortex-wide dynamics with wide-field calcium imaging. J. Neurosci. 41, 4160–4168 (2021). 33893217 10.1523/JNEUROSCI.3003-20.2021 PMC8143209

[7] Kim T H. & Schnitzer, M. J. Fluorescence imaging of large-scale neural ensemble dynamics. Cell 185, 9–41 (2022). 34995519 10.1016/j.cell.2021.12.007 PMC8849612

[8] Urai AE, Doiron B, Leifer AM & Churchland AK Large-scale neural recordings call for new insights to link brain and behavior. Nat. Neurosci. 25, 11–19 (2022). 34980926 10.1038/s41593-021-00980-9

[9] Siegel M, Buschman TJ & Miller EK Cortical information flow during flexible sensorimotor decisions. Science 348, 1352–1355 (2015). 26089513 10.1126/science.aab0551 PMC4721574

[10] Hernández A Procedure for recording the simultaneous activity of single neurons distributed across cortical areas during sensory discrimination. Proc Natl Acad. Sci. USA 105, 16785–16790 (2008). 18946031 10.1073/pnas.0808702105 PMC2571910

[11] Hernández A Decoding a perceptual decision process across cortex. Neuron 66, 300–314 (2010). 20435005 10.1016/j.neuron.2010.03.031

[12] Paulk AC Large-scale neural recordings with single neuron resolution using Neuropixels probes in human cortex. Nat. Neurosci. 25, 252–263 (2022). 35102333 10.1038/s41593-021-00997-0

[13] Oh SW A mesoscale connectome of the mouse brain. Nature 508, 207–214 (2014). 24695228 10.1038/nature13186 PMC5102064

[14] Han Y The logic of single-cell projections from visual cortex. Nature 556, 51–56 (2018). 29590093 10.1038/nature26159 PMC6585423

[15] Brown CE, Aminoltejari K, Erb H, Winship IR & Murphy TH In vivo voltage-sensitive dye imaging in adult mice reveals that somatosensory maps lost to stroke are replaced over weeks by new structural and functional circuits with prolonged modes of activation within both the peri-infarct zone and distant sites. J. Neurosci. 29, 1719–1734 (2009). 19211879 10.1523/JNEUROSCI.4249-08.2009 PMC6666293

[16] Ferezou I Spatiotemporal dynamics of cortical sensorimotor integration in behaving mice. Neuron 56, 907–923 (2007). 18054865 10.1016/j.neuron.2007.10.007

[17] Santos L, Opris I, Fuqua J, Hampson RE & Deadwyler SA A novel tetrode microdrive for simultaneous multi-neuron recording from different regions of primate brain. J. Neurosci. Methods 205, 368–374 (2012). 22326226 10.1016/j.jneumeth.2012.01.006 PMC3342772

[18] Allen WE Thirst regulates motivated behavior through modulation of brainwide neural population dynamics. Science 364, eaav3932 (2019). 10.1126/science.aav3932 PMC6711472 30948440

[19] Engel T A. & Steinmetz, N. A. New perspectives on dimensionality and variability from large-scale cortical dynamics. Curr. Opin. Neurobiol. 58, 181–190 (2019). 31585331 10.1016/j.conb.2019.09.003 PMC6859189

[20] Schneider DM Reflections of action in sensory cortex. Curr. Opin. Neurobiol. 64, 53–59 (2020). 32171079 10.1016/j.conb.2020.02.004

[21] Musall S, Kaufman MT, Juavinett AL, Gluf S & Churchland AK Single-trial neural dynamics are dominated by richly varied movements. Nat. Neurosci. 22, 1677–1686 (2019). 31551604 10.1038/s41593-019-0502-4 PMC6768091

[22] Stringer C Spontaneous behaviors drive multidimensional, brainwide activity. Science 364, eaav7893 (2019). 10.1126/science.aav7893 PMC6525101 31000656

[23] Kauvar IV Cortical observation by synchronous multifocal optical sampling reveals widespread population encoding of actions. Neuron 107, 351–367.e19 (2020). 32433908 10.1016/j.neuron.2020.04.023 PMC7687350

[24] Schneider DM, Nelson A & Mooney R A synaptic and circuit basis for corollary discharge in the auditory cortex. Nature 513, 189–194 (2014). 25162524 10.1038/nature13724 PMC4248668

[25] Saxena S & Cunningham J P Towards the neural population doctrine. Curr Opin. Neurobiol. 55, 103–111 (2019). 30877963 10.1016/j.conb.2019.02.002

[26] Dong HW The Allen Reference Atlas: A Digital Color Brain Atlas of the C57Bl/6J Male Mouse (Wiley, 2008).

[27] Paxinos G & Franklin KB J. Paxinos and Franklin’s the Mouse Brain in Stereotaxic Coordinates (Academic, 2019).

[28] Swanson L Brain Maps: Structure of the Rat Brain (Gulf Professional Publishing, 2004).

[29] Zingg B Neural networks of the mouse neocortex. Cell 10.1016/j.cell.2014.02.023 (2014). PMC4169118 24581503

[30] Jones AR, Overly CC & Sunkin SM The Allen Brain Atlas: 5 years and beyond. Nat. Rev. Neurosci. 10, 821–828 (2009). 19826436 10.1038/nrn2722

[31] Lein ES Genome-wide atlas of gene expression in the adult mouse brain. Nature 445, 168 (2007). 17151600 10.1038/nature05453

[32] Wang Q The Allen mouse brain common coordinate framework: a 3D reference atlas. Cell 181, 936–953.e20 (2020). 32386544 10.1016/j.cell.2020.04.007 PMC8152789

[33] Siegle JH Survey of spiking in the mouse visual system reveals functional hierarchy. Nature 592, 86–92 (2021). 33473216 10.1038/s41586-020-03171-x PMC10399640

[34] Steinmetz NA, Zatka-Haas P, Carandini M & Harris KD Distributed coding of choice, action and engagement across the mouse brain. Nature 576, 266–273 (2019). 31776518 10.1038/s41586-019-1787-x PMC6913580

[35] Hafting T, Fyhn M, Molden S, Moser M-B & Moser EI Microstructure of a spatial map in the entorhinal cortex. Nature 436, 801–806 (2005). 15965463 10.1038/nature03721

[36] da Silva JA, Tecuapetla F, Paixão V & Costa RM Dopamine neuron activity before action initiation gates and invigorates future movements. Nature 554, 244–248 (2018). 29420469 10.1038/nature25457

[37] Carus-Cadavieco M Gamma oscillations organize top-down signalling to hypothalamus and enable food seeking. Nature 542, 232–236 (2017). 28146472 10.1038/nature21066

[38] Senzai Y, Fernandez-Ruiz A & Buzsáki G Layer-specific physiological features and interlaminar interactions in the primary visual cortex of the mouse. Neuron 101, 500–513.e5 (2019). 30635232 10.1016/j.neuron.2018.12.009 PMC6367010

[39] Couto J Chronic, cortex-wide imaging of specific cell populations during behavior. Nat. Protoc. 16, 3241–3263 (2021). 34075229 10.1038/s41596-021-00527-z PMC8788140

[40] Dockès J NeuroQuery, comprehensive meta-analysis of human brain mapping. eLife 9, e53385 (2020). 32129761 10.7554/eLife.53385 PMC7164961

[41] Yarkoni T, Poldrack RA, Nichols TE, Van Essen DC & Wager TD Large-scale automated synthesis of human functional neuroimaging data. Nat. Methods 8, 665–670 (2011). 21706013 10.1038/nmeth.1635 PMC3146590

[42] Kim CK, Adhikari A & Deisseroth K Integration of optogenetics with complementary methodologies in systems neuroscience. Nat. Rev. Neurosci. 18, 222–235 (2017). 28303019 10.1038/nrn.2017.15 PMC5708544

[43] Tervo DGR A designer AAV variant permits efficient retrograde access to projection neurons. Neuron 92, 372–382 (2016). 27720486 10.1016/j.neuron.2016.09.021 PMC5872824

[44] Fenno LE Targeting cells with single vectors using multiple-feature Boolean logic. Nat. Methods 11, 763–772 (2014). 24908100 10.1038/nmeth.2996 PMC4085277

[45] Andalman AS Neuronal dynamics regulating brain and behavioral state transitions. Cell 177, 970–985.e20 (2019). 31031000 10.1016/j.cell.2019.02.037 PMC6726130

[46] Lovett-Barron M Ancestral circuits for the coordinated modulation of brain state. Cell 171, 1411–1423.e17 (2017). 29103613 10.1016/j.cell.2017.10.021 PMC5725395

[47] Xu S Behavioral state coding by molecularly defined paraventricular hypothalamic cell type ensembles. Science 370, eabb2494 (2020). 33060330 10.1126/science.abb2494 PMC11938375

[48] Allen WE Global representations of goal-directed behavior in distinct cell types of mouse neocortex. Neuron 94, 891–907.e6 (2017). 28521139 10.1016/j.neuron.2017.04.017 PMC5723385

[49] Chan KY Engineered AAVs for efficient noninvasive gene delivery to the central and peripheral nervous systems. Nat. Neurosci. 20, 1172–1179 (2017). 28671695 10.1038/nn.4593 PMC5529245

[50] Lima SQ, Hromádka T, Znamenskiy P & Zador AM PINP: a new method of tagging neuronal populations for identification during in vivo electrophysiological recording. PLoS ONE 4, e6099 (2009). 19584920 10.1371/journal.pone.0006099 PMC2702752

[51] Wolff SBE Amygdala interneuron subtypes control fear learning through disinhibition. Nature 509, 453–458 (2014). 24814341 10.1038/nature13258

[52] Cohen JY, Haesler S, Vong L, Lowell BB & Uchida N Neuron-type-specific signals for reward and punishment in the ventral tegmental area. Nature 482, 85–88 (2012). 22258508 10.1038/nature10754 PMC3271183

[53] Herrera CG Hypothalamic feedforward inhibition of thalamocortical network controls arousal and consciousness. Nat. Neurosci. 19, 290–298 (2016). 26691833 10.1038/nn.4209 PMC5818272

[54] Juavinett AL, Bekheet G & Churchland AK Chronically implanted Neuropixels probes enable high-yield recordings in freely moving mice. eLife 8, e47188 (2019). 31411559 10.7554/eLife.47188 PMC6707768

[55] Schoonover CE, Ohashi SN, Axel R & Fink AJP Representational drift in primary olfactory cortex. Nature 594, 541–546 (2021). 34108681 10.1038/s41586-021-03628-7

[56] Steinmetz NA Neuropixels 2.0: a miniaturized high-density probe for stable, long-term brain recordings. Science 372, eabf4588 (2021). 33859006 10.1126/science.abf4588 PMC8244810

[57] Chung JE High-density, long-lasting, and multi-region electrophysiological recordings using polymer electrode arrays. Neuron 101, 21–31.e5 (2019). 30502044 10.1016/j.neuron.2018.11.002 PMC6326834

[58] Cardin JA, Crair MC & Higley MJ Mesoscopic imaging: shining a wide light on large-scale neural dynamics. Neuron 108, 33–43 (2020). 33058764 10.1016/j.neuron.2020.09.031 PMC7577373

[59] Grinvald A & Hildesheim R VSDI: a new era in functional imaging of cortical dynamics. Nat. Rev. Neurosci. 5, 874–885 (2004). 15496865 10.1038/nrn1536

[60] Mohajerani MH Spontaneous cortical activity alternates between motifs defined by regional axonal projections. Nat. Neurosci. 16, 1426–1435 (2013). 23974708 10.1038/nn.3499 PMC3928052

[61] Dana H Thy1-GCaMP6 transgenic mice for neuronal population imaging in vivo. PLoS ONE 9, e108697 (2014). 25250714 10.1371/journal.pone.0108697 PMC4177405

[62] Madisen L Transgenic mice for intersectional targeting of neural sensors and effectors with high specificity and performance. Neuron 85, 942–958 (2015). 25741722 10.1016/j.neuron.2015.02.022 PMC4365051

[63] Wekselblatt JB, Flister ED, Piscopo DM & Niell CM Large-scale imaging of cortical dynamics during sensory perception and behavior. J. Neurophysiol. 115, 2852–2866 (2016). 26912600 10.1152/jn.01056.2015 PMC4922607

[64] Deverman BE Cre-dependent selection yields AAV variants for widespread gene transfer to the adult brain. Nat. Biotechnol. 34, 204–209 (2016). 26829320 10.1038/nbt.3440 PMC5088052

[65] Guo ZV Flow of cortical activity underlying a tactile decision in mice. Neuron 81, 179–194 (2014). 24361077 10.1016/j.neuron.2013.10.020 PMC3984938

[66] Ratzlaff EH & Grinvald A A tandem-lens epifluorescence macroscope: hundred-fold brightness advantage for wide-field imaging. J. Neurosci. Methods 36, 127–137 (1991). 1905769 10.1016/0165-0270(91)90038-2

[67] Kim CK Simultaneous fast measurement of circuit dynamics at multiple sites across the mammalian brain. Nat. Methods 13, 325–328 (2016). 26878381 10.1038/nmeth.3770 PMC5717315

[68] Valley MT Separation of hemodynamic signals from GCaMP fluorescence measured with wide-field imaging. J. Neurophysiol. 123, 356–366 (2020). 31747332 10.1152/jn.00304.2019

[69] Daigle TL A suite of transgenic driver and reporter mouse lines with enhanced brain-cell-type targeting and functionality. Cell 174, 465–480.e22 (2018). 30007418 10.1016/j.cell.2018.06.035 PMC6086366

[70] Gerfen CR, Paletzki R & Heintz N GENSAT BAC Cre-recombinase driver lines to study the functional organization of cerebral cortical and basal ganglia circuits. Neuron 80, 1368–1383 (2013). 24360541 10.1016/j.neuron.2013.10.016 PMC3872013

[71] Matho KS Genetic dissection of the glutamatergic neuron system in cerebral cortex. Nature 598, 182–187 (2021). 34616069 10.1038/s41586-021-03955-9 PMC8494647

[72] Callaway EM A multimodal cell census and atlas of the mammalian primary motor cortex. Nature 598, 86–102 (2021). 34616075 10.1038/s41586-021-03950-0 PMC8494634

[73] Fenno LE Comprehensive dual- and triple-feature intersectional single-vector delivery of diverse functional payloads to cells of behaving mammals. Neuron 107, 836–853.e11 (2020). 32574559 10.1016/j.neuron.2020.06.003 PMC7687746

[74] Harris J Anatomical characterization of Cre driver mice for neural circuit mapping and manipulation. Front. Neural Circuits 10.3389/fncir.2014.00076 (2014). PMC4091307 25071457

[75] Waters J Sources of widefield fluorescence from the brain. eLife 9, e59841 (2020). 33155981 10.7554/eLife.59841 PMC7647397

[76] Lohani S Dual color mesoscopic imaging reveals spatiotemporally heterogeneous coordination of cholinergic and neocortical activity. Preprint at bioRxiv 10.1101/2020.12.09.418632 (2020). PMC10661869 36443609

[77] Sabatini BL & Tian L Imaging neurotransmitter and neuromodulator dynamics in vivo with genetically encoded indicators. Neuron 108, 17–32 (2020). 33058762 10.1016/j.neuron.2020.09.036

[78] Shen Y, Nasu Y, Shkolnikov I, Kim A & Campbell RE Engineering genetically encoded fluorescent indicators for imaging of neuronal activity: progress and prospects. Neurosci. Res. 152, 3–14 (2020). 31991206 10.1016/j.neures.2020.01.011

[79] Steinmetz NA Aberrant cortical activity in multiple GCaMP6-expressing transgenic mouse lines. eNeuro 4, ENEURO.0207-17.2017 (2017). 10.1523/ENEURO.0207-17.2017 PMC5604087 28932809

[80] Heffley W Coordinated cerebellar climbing fiber activity signals learned sensorimotor predictions. Nat. Neurosci. 21, 1431–1441 (2018). 30224805 10.1038/s41593-018-0228-8 PMC6362851

[81] Ackman JB, Burbridge TJ & Crair MC Retinal waves coordinate patterned activity throughout the developing visual system. Nature 490, 219–225 (2012). 23060192 10.1038/nature11529 PMC3962269

[82] Li Y, Turan Z & Meister M Functional architecture of motion direction in the mouse superior colliculus. Curr. Biol. 30, 3304–3315.e4 (2020). 32649907 10.1016/j.cub.2020.06.023 PMC8221388

[83] Scott BB Imaging cortical dynamics in GCaMP transgenic rats with a head-mounted widefield macroscope. Neuron 100, 1045–1058.e5 (2018). 30482694 10.1016/j.neuron.2018.09.050 PMC6283673

[84] Rynes ML Miniaturized head-mounted microscope for whole-cortex mesoscale imaging in freely behaving mice. Nat. Methods 18, 417–425 (2021). 33820987 10.1038/s41592-021-01104-8 PMC8034419

[85] Lake EMR Simultaneous cortex-wide fluorescence Ca^2+^ imaging and whole-brain fMRI. Nat. Methods 17, 1262–1271 (2020). 33139894 10.1038/s41592-020-00984-6 PMC7704940

[86] Murphy TH High-throughput automated home-cage mesoscopic functional imaging of mouse cortex. Nat. Commun. 7, 11611 (2016). 27291514 10.1038/ncomms11611 PMC4909937

[87] Murphy TH Automated task training and longitudinal monitoring of mouse mesoscale cortical circuits using home cages. eLife 9, e55964 (2020). 32412409 10.7554/eLife.55964 PMC7332290

[88] Kim TH Long-term optical access to an estimated one million neurons in the live mouse cortex. Cell Rep. 17, 3385–3394 (2016). 28009304 10.1016/j.celrep.2016.12.004 PMC5459490

[89] Ghanbari L Cortex-wide neural interfacing via transparent polymer skulls. Nat. Commun. 10, 1500 (2019). 30940809 10.1038/s41467-019-09488-0 PMC6445105

[90] Freeman J Mapping brain activity at scale with cluster computing. Nat. Methods 11, 941–950 (2014). 25068736 10.1038/nmeth.3041

[91] Kim CK Prolonged, brain-wide expression of nuclear-localized GCaMP3 for functional circuit mapping. Front. Neural Circuits 8, 138 (2014). 25505384 10.3389/fncir.2014.00138 PMC4244806

[92] Vladimirov N Light-sheet functional imaging in fictively behaving zebrafish. Nat. Methods 11, 883–884 (2014). 25068735 10.1038/nmeth.3040

[93] Chen Y Soma-targeted imaging of neural circuits by ribosome tethering. Neuron 107, 454–469.e6 (2020). 32574560 10.1016/j.neuron.2020.05.005 PMC7540731

[94] Shemesh OA Precision calcium imaging of dense neural populations via a cell-body-targeted calcium indicator. Neuron 107, 470–486.e11 (2020). 32592656 10.1016/j.neuron.2020.05.029 PMC7415598

[95] Lim ST, Antonucci DE, Scannevin RH & Trimmer JS A novel targeting signal for proximal clustering of the Kv2.1 K^+^ channel in hippocampal neurons. Neuron 25, 385–397 (2000). 10719893 10.1016/s0896-6273(00)80902-2

[96] Cramer SW Through the looking glass: a review of cranial window technology for optical access to the brain. J. Neurosci. Methods 354, 109100 (2021). 33600850 10.1016/j.jneumeth.2021.109100 PMC8100903

[97] Fan J Video-rate imaging of biological dynamics at centimetre scale and micrometre resolution. Nat. Photonics 13, 809–816 (2019).

[98] Broxton M Wave optics theory and 3-D deconvolution for the light field microscope. Opt. Express 21, 25418–25439 (2013). 24150383 10.1364/OE.21.025418 PMC3867103

[99] Nöbauer T Video rate volumetric Ca_2+_ imaging across cortex using seeded iterative demixing (SID) microscopy. Nat. Methods 14, 811–818 (2017). 28650477 10.1038/nmeth.4341

[100] Voleti V Real-time volumetric microscopy of in vivo dynamics and large-scale samples with SCAPE 2.0. Nat. Methods 16, 1054–1062 (2019). 31562489 10.1038/s41592-019-0579-4 PMC6885017

[101] Chang C.-P (Jonathan) & Holy TE in Optical Techniques in Neurosurgery, Neurophotonics, and Optogenetics vol. 11629, 20–28 (SPIE, 2021).

[102] Kumar M, Kishore S, Nasenbeny J, McLean DL & Kozorovitskiy Y Integrated one- and two-photon scanned oblique plane illumination (SOPi) microscopy for rapid volumetric imaging. Opt. Express 26, 13027–13041 (2018). 29801336 10.1364/OE.26.013027 PMC6005676

[103] Xue Y, Davison IG, Boas DA & Tian L Single-shot 3D wide-field fluorescence imaging with a Computational Miniature Mesoscope. Sci. Adv. 6, eabb7508 (2020). 33087364 10.1126/sciadv.abb7508 PMC7577725

[104] Ebrahimi S Emergent reliability in sensory cortical coding and inter-area communication. Nature 10.1038/s41586-022-04724-y (2022). PMC10985415 35589841

[105] Gunaydin LA Natural neural projection dynamics underlying social behavior. Cell 157, 1535–1551 (2014). 24949967 10.1016/j.cell.2014.05.017 PMC4123133

[106] Cui G Concurrent activation of striatal direct and indirect pathways during action initiation. Nature 494, 238–242 (2013). 23354054 10.1038/nature11846 PMC4039389

[107] Lütcke H Optical recording of neuronal activity with a genetically-encoded calcium indicator in anesthetized and freely moving mice. Front. Neural Circuits 4, 9 (2010). 20461230 10.3389/fncir.2010.00009 PMC2866455

[108] Schulz K Simultaneous BOLD fMRI and fiberoptic calcium recording in rat neocortex. Nat. Methods 9, 597–602 (2012). 22561989 10.1038/nmeth.2013

[109] Stroh A Making waves: initiation and propagation of corticothalamic Ca^2+^ waves in vivo. Neuron 77, 1136–1150 (2013). 23522048 10.1016/j.neuron.2013.01.031

[110] Pisanello M The three-dimensional signal collection field for fiber photometry in brain tissue. Front. Neurosci. 13, 82 (2019). 30863275 10.3389/fnins.2019.00082 PMC6399578

[111] Marshall JD Cell-type-specific optical recording of membrane voltage dynamics in freely moving mice. Cell 167, 1650–1662.e15 (2016). 27912066 10.1016/j.cell.2016.11.021 PMC5382987

[112] Pisano F Depth-resolved fiber photometry with a single tapered optical fiber implant. Nat. Methods 16, 1185–1192 (2019). 31591577 10.1038/s41592-019-0581-x

[113] Sych Y, Chernysheva M, Sumanovski LT & Helmchen F High-density multi-fiber photometry for studying large-scale brain circuit dynamics. Nat. Methods 16, 553–560 (2019). 31086339 10.1038/s41592-019-0400-4

[114] Pnevmatikakis EA Simultaneous denoising, deconvolution, and demixing of calcium imaging data. Neuron 89, 285–299 (2016). 26774160 10.1016/j.neuron.2015.11.037 PMC4881387

[115] Siegle JH Reconciling functional differences in populations of neurons recorded with two-photon imaging and electrophysiology. eLife 10, e69068 (2021). 34270411 10.7554/eLife.69068 PMC8285106

[116] Wei Z A comparison of neuronal population dynamics measured with calcium imaging and electrophysiology. PLoS Comput. Biol. 16, e1008198 (2020). 32931495 10.1371/journal.pcbi.1008198 PMC7518847

[117] Abdelfattah AS Bright and photostable chemigenetic indicators for extended in vivo voltage imaging. Science 365, 699–704 (2019). 31371562 10.1126/science.aav6416

[118] Jin L Single action potentials and subthreshold electrical events imaged in neurons with a fluorescent protein voltage probe. Neuron 75, 779–785 (2012). 22958819 10.1016/j.neuron.2012.06.040 PMC3439164

[119] Lin MZ & Schnitzer MJ Genetically encoded indicators of neuronal activity. Nat. Neurosci. 19, 1142–1153 (2016). 27571193 10.1038/nn.4359 PMC5557009

[120] Piatkevich KD Population imaging of neural activity in awake behaving mice. Nature 574, 413–417 (2019). 31597963 10.1038/s41586-019-1641-1 PMC6858559

[121] Xu Y, Zou P & Cohen AE Voltage imaging with genetically encoded indicators. Curr. Opin. Chem. Biol. 39, 1–10 (2017). 28460291 10.1016/j.cbpa.2017.04.005 PMC5581692

[122] Villette V Ultrafast two-photon imaging of a high-gain voltage indicator in awake behaving mice. Cell 179, 1590–1608.e23 (2019). 31835034 10.1016/j.cell.2019.11.004 PMC6941988

[123] Adam Y Voltage imaging and optogenetics reveal behaviour-dependent changes in hippocampal dynamics. Nature 569, 413–417 (2019). 31043747 10.1038/s41586-019-1166-7 PMC6613938

[124] Fan LZ All-optical synaptic electrophysiology probes mechanism of ketamine-induced disinhibition. Nat. Methods 15, 823–831 (2018). 30275587 10.1038/s41592-018-0142-8 PMC6204345

[125] Fan LZ All-optical electrophysiology reveals the role of lateral inhibition in sensory processing in cortical layer 1. Cell 180, 521–535.e18 (2020). 31978320 10.1016/j.cell.2020.01.001 PMC7259440

[126] Hochbaum DR All-optical electrophysiology in mammalian neurons using engineered microbial rhodopsins. Nat. Methods 11, 825–833 (2014). 24952910 10.1038/nmeth.3000 PMC4117813

[127] Piatkevich KD A robotic multidimensional directed evolution approach applied to fluorescent voltage reporters. Nat. Chem. Biol. 14, 352–360 (2018). 29483642 10.1038/s41589-018-0004-9 PMC5866759

[128] Marshel JH Cortical layer–specific critical dynamics triggering perception. Science 365, eaaw5202 (2019). 31320556 10.1126/science.aaw5202 PMC6711485

[129] Wu J Kilohertz two-photon fluorescence microscopy imaging of neural activity in vivo. Nat. Methods 17, 287–290 (2020). 32123392 10.1038/s41592-020-0762-7 PMC7199528

[130] Carandini M Imaging the awake visual cortex with a genetically encoded voltage indicator. J. Neurosci. 35, 53–63 (2015). 25568102 10.1523/JNEUROSCI.0594-14.2015 PMC4287159

[131] Platisa J Voltage imaging using transgenic mouse lines expressing the GEVI ArcLight in two olfactory cell types. Preprint at bioRxiv 10.1101/2020.08.26.268904 (2020).

[132] Platisa J High-speed low-light in vivo two-photon voltage imaging of large neuronal populations. Preprint at bioRxiv 10.1101/2021.12.07.471668 (2021). PMC10894646 36973547

[133] Gottschalk S Rapid volumetric optoacoustic imaging of neural dynamics across the mouse brain. Nat. Biomed. Eng. 3, 392–401 (2019). 30992553 10.1038/s41551-019-0372-9 PMC6825512

[134] Rabut C Ultrasound technologies for imaging and modulating neural activity. Neuron 108, 93–110 (2020). 33058769 10.1016/j.neuron.2020.09.003 PMC7577369

[135] Helmchen F & Denk W Deep tissue two-photon microscopy. Nat. Methods 2, 932–940 (2005). 16299478 10.1038/nmeth818

[136] Lecoq J Visualizing mammalian brain area interactions by dual-axis two-photon calcium imaging. Nat. Neurosci. 17, 1825–1829 (2014). 25402858 10.1038/nn.3867 PMC5289313

[137] Wagner MJ Shared cortex-cerebellum dynamics in the execution and learning of a motor task. Cell 177, 669–682.e24 (2019). 30929904 10.1016/j.cell.2019.02.019 PMC6500577

[138] Sofroniew NJ, Flickinger D, King J & Svoboda K A large field of view two-photon mesoscope with subcellular resolution for in vivo imaging. eLife 5, e14472 (2016). 27300105 10.7554/eLife.14472 PMC4951199

[139] Stirman JN, Smith IT, Kudenov MW & Smith SL Wide field-of-view, multi-region two-photon imaging of neuronal activity in the mammalian brain. Nat. Biotechnol. 34, 857–862 (2016). 27347754 10.1038/nbt.3594 PMC4980167

[140] Chen JL, Voigt FF, Javadzadeh M, Krueppel R & Helmchen F Long-range population dynamics of anatomically defined neocortical networks. eLife 5, e14679 (2016). 27218452 10.7554/eLife.14679 PMC4929001

[141] Condylis C Context-dependent sensory processing across primary and secondary somatosensory cortex. Neuron 106, 515–525.e5 (2020). 32164873 10.1016/j.neuron.2020.02.004 PMC7210055

[142] Yu C-H, Stirman JN, Yu Y, Hira R & Smith SL Diesel2p mesoscope with dual independent scan engines for flexible capture of dynamics in distributed neural circuitry. Nat. Commun. 12, 6639 (2021). 34789723 10.1038/s41467-021-26736-4 PMC8599518

[143] Clough M Flexible simultaneous mesoscale two-photon imaging of neural activity at high speeds. Nat. Commun. 12, 6638 (2021). 34789730 10.1038/s41467-021-26737-3 PMC8599611

[144] Lu R Rapid mesoscale volumetric imaging of neural activity with synaptic resolution. Nat. Methods 17, 291–294 (2020). 32123393 10.1038/s41592-020-0760-9 PMC7192636

[145] Demas J High-speed, cortex-wide volumetric recording of neuroactivity at cellular resolution using light beads microscopy. Nat. Methods 18, 1103–1111 (2021). 34462592 10.1038/s41592-021-01239-8 PMC8958902

[146] Rumyantsev OI Fundamental bounds on the fidelity of sensory cortical coding. Nature 580, 100–105 (2020). 32238928 10.1038/s41586-020-2130-2

[147] Zhang T Kilohertz two-photon brain imaging in awake mice. Nat. Methods 16, 1119–1122 (2019). 31659327 10.1038/s41592-019-0597-2 PMC9438750

[148] Yang SJ Extended field-of-view and increased-signal 3D holographic illumination with time-division multiplexing. Opt. Express 23, 32573 (2015). 26699047 10.1364/OE.23.032573 PMC4775739

[149] Barson D Simultaneous mesoscopic and two-photon imaging of neuronal activity in cortical circuits. Nat. Methods 17, 107–113 (2020). 31686040 10.1038/s41592-019-0625-2 PMC6946863

[150] Zong W Large-scale two-photon calcium imaging in freely moving mice. Cell 185, 1240–1256.e30 (2021). 10.1016/j.cell.2022.02.017 PMC8970296 35305313

[151] Jun JJ Fully integrated silicon probes for high-density recording of neural activity. Nature 551, 232–236 (2017). 29120427 10.1038/nature24636 PMC5955206

[152] Voigts J, Newman JP, Wilson MA & Harnett MT An easy-to-assemble, robust, and lightweight drive implant for chronic tetrode recordings in freely moving animals. J. Neural Eng. 17, 026044 (2020). 32074511 10.1088/1741-2552/ab77f9 PMC8878001

[153] Varol E Decentralized motion inference and registration of neuropixel data. In ICASSP 2021–2021 IEEE International Conference on Acoustics, Speech and Signal Processing (ICASSP) 1085–1089 (IEEE, 2021).

[154] Luo TZ An approach for long-term, multi-probe Neuropixels recordings in unrestrained rats. eLife 9, e59716 (2020). 33089778 10.7554/eLife.59716 PMC7721443

[155] Jensen KHR & Berg RW CLARITY-compatible lipophilic dyes for electrode marking and neuronal tracing. Sci. Rep. 6, 32674 (2016). 27597115 10.1038/srep32674 PMC5011694

[156] Vázquez-Guardado A, Yang Y, Bandodkar AJ & Rogers JA Recent advances in neurotechnologies with broad potential for neuroscience research. Nat. Neurosci. 23, 1522–1536 (2020). 33199897 10.1038/s41593-020-00739-8

[157] Wang X A parylene neural probe array for multiregion deep brain recordings. J. Microelectromech. Syst. 29, 499–513 (2020). 35663261 10.1109/jmems.2020.3000235 PMC9164222

[158] Liu X Multimodal neural recordings with Neuro-FITM uncover diverse patterns of cortical–hippocampal interactions. Nat. Neurosci. 24, 886–896 (2021). 33875893 10.1038/s41593-021-00841-5 PMC8627685

[159] Clancy KB, Orsolic I & Mrsic-Flogel TD Locomotion-dependent remapping of distributed cortical networks. Nat. Neurosci. 22, 778–786 (2019). 30858604 10.1038/s41593-019-0357-8 PMC6701985

[160] Peters AJ, Fabre JMJ, Steinmetz NA, Harris KD & Carandini M Striatal activity topographically reflects cortical activity. Nature 591, 420–425 (2021). 33473213 10.1038/s41586-020-03166-8 PMC7612253

[161] Kleinfeld D Can one concurrently record electrical spikes from every neuron in a mammalian brain? Neuron 103, 1005–1015 (2019). 31495645 10.1016/j.neuron.2019.08.011 PMC6763354

[162] Trautmann E Accurate estimation of neural population dynamics without spike sorting. Neuron 103, 292–308.e4 (2019). 31171448 10.1016/j.neuron.2019.05.003 PMC7002296

[163] Ecker AS Decorrelated neuronal firing in cortical microcircuits. Science 327, 584–587 (2010). 20110506 10.1126/science.1179867

[164] Renart A The asynchronous state in cortical circuits. Science 327, 587–590 (2010). 20110507 10.1126/science.1179850 PMC2861483

[165] Dacre J A cerebellar-thalamocortical pathway drives behavioral context-dependent movement initiation. Neuron 109, 2326–2338.e8 (2021). 34146469 10.1016/j.neuron.2021.05.016 PMC8315304

[166] Wang W Coordination of escape and spatial navigation circuits orchestrates versatile flight from threats. Neuron 109, 1848–1860.e8 (2021). 33861942 10.1016/j.neuron.2021.03.033 PMC8178241

[167] Adrian ED The impulses produced by sensory nerve endings. J. Physiol. 61, 49–72 (1926). 16993776 10.1113/jphysiol.1926.sp002273 PMC1514809

[168] Sherrington C The Integrative Action of the Nervous System (Charles Scribner’s Sons, 1906).

[169] Hubel DH & Wiesel TN Receptive fields of single neurones in the cat’s striate cortex. J. Physiol. 148, 574–591 (1959). 14403679 10.1113/jphysiol.1959.sp006308 PMC1363130

[170] Chichilnisky EJ A simple white noise analysis of neuronal light responses. Netw. Comput. Neural Syst. 12, 199–213 (2001). 11405422

[171] Schwartz O, Pillow JW, Rust NC & Simoncelli EP Spike-triggered neural characterization. J. Vis. 6, 13–13 (2006). 10.1167/6.4.13 16889482

[172] Pillow JW Spatio-temporal correlations and visual signalling in a complete neuronal population. Nature 454, 995–999 (2008). 18650810 10.1038/nature07140 PMC2684455

[173] Truccolo W, Eden UT, Fellows MR, Donoghue JP & Brown EN A point process framework for relating neural spiking activity to spiking history, neural ensemble, and extrinsic covariate effects. J. Neurophysiol. 93, 1074–1089 (2005). 15356183 10.1152/jn.00697.2004

[174] Kang B & Druckmann S Approaches to inferring multi-regional interactions from simultaneous population recordings. Curr. Opin. Neurobiol. 65, 108–119 (2020). 33227602 10.1016/j.conb.2020.10.004 PMC7853322

[175] Keeley SL, Zoltowski DM, Aoi MC & Pillow JW Modeling statistical dependencies in multi-region spike train data. Curr. Opin. Neurobiol. 65, 194–202 (2020). 33334641 10.1016/j.conb.2020.11.005 PMC7769979

[176] Yates JL, Park IM, Katz LN, Pillow JW & Huk AC Functional dissection of signal and noise in MT and LIP during decision-making. Nat. Neurosci. 20, 1285–1292 (2017). 28758998 10.1038/nn.4611 PMC5673485

[177] Benjamin AS Modern machine learning as a benchmark for fitting neural responses. Front. Comput. Neurosci. 12, 56 (2018). 30072887 10.3389/fncom.2018.00056 PMC6060269

[178] Linderman S, Adams RP & Pillow JW Bayesian latent structure discovery from multi-neuron recordings. in Advances in Neural Information Processing Systems Vol. 29 (NIPS, 2016).

[179] Pedregosa F Scikit-learn: machine learning in python. J. Mach. Learn. Res. 12, 2825–2830 (2011).

[180] Vesuna S Deep posteromedial cortical rhythm in dissociation. Nature 586, 87–94 (2020). 32939091 10.1038/s41586-020-2731-9 PMC7553818

[181] Harris KD Nonsense correlations in neuroscience. Preprint at bioRxiv 10.1101/2020.11.29.402719 (2021).

[182] Meijer G Neurons in the mouse brain correlate with cryptocurrency price: a cautionary tale. Preprint at PsyArXiv 10.31234/osf.io/fa4wz (2021).

[183] Zagha E The importance of accounting for movement when relating neuronal activity to sensory and cognitive processes. J. Neurosci. 42, 1375–1382 (2022). 35027407 10.1523/JNEUROSCI.1919-21.2021 PMC8883841

[184] Cunningham JP & Yu BM Dimensionality reduction for large-scale neural recordings. Nat. Neurosci. 17, 1500–1509 (2014). 25151264 10.1038/nn.3776 PMC4433019

[185] Churchland MM Neural population dynamics during reaching. Nature 487, 51–56 (2012). 22722855 10.1038/nature11129 PMC3393826

[186] Gao P A theory of multineuronal dimensionality, dynamics and measurement. Preprint at bioRxiv 10.1101/214262 (2017).

[187] Stringer C, Pachitariu M, Steinmetz N, Carandini M & Harris KD High-dimensional geometry of population responses in visual cortex. Nature 571, 361–365 (2019). 31243367 10.1038/s41586-019-1346-5 PMC6642054

[188] Yu BM Gaussian-process factor analysis for low-dimensional single-trial analysis of neural population activity. J. Neurophysiol. 102, 614–635 (2009). 19357332 10.1152/jn.90941.2008 PMC2712272

[189] Gallego JA, Perich MG, Chowdhury RH, Solla SA & Miller LE Long-term stability of cortical population dynamics underlying consistent behavior. Nat. Neurosci. 23, 260–270 (2020). 31907438 10.1038/s41593-019-0555-4 PMC7007364

[190] Linderman S Bayesian learning and inference in recurrent switching linear dynamical systems. in Proceedings of the 20th International Conference on Artificial Intelligence and Statistics 914–922 (PMLR, 2017).

[191] Linderman S, Nichols A, Blei D, Zimmer M & Paninski L Hierarchical recurrent state space models reveal discrete and continuous dynamics of neural activity in C. elegans. Preprint at bioRxiv 10.1101/621540 (2019).

[192] Humphries MD Strong and weak principles of neural dimension reduction. Preprint at 10.48550/arXiv.2011.08088 (2021).

[193] Gollisch T & Meister M Rapid neural coding in the retina with relative spike latencies. Science 319, 1108–1111 (2008). 18292344 10.1126/science.1149639

[194] Maimon G & Assad JA Beyond Poisson: increased spike-time regularity across primate parietal cortex. Neuron 62, 426–440 (2009). 19447097 10.1016/j.neuron.2009.03.021 PMC2743683

[195] Vyas S, Golub MD, Sussillo D & Shenoy KV Computation through neural population dynamics. Annu. Rev. Neurosci. 43, 249–275 (2020). 32640928 10.1146/annurev-neuro-092619-094115 PMC7402639

[196] Ames KC, Ryu SI & Shenoy KV Neural dynamics of reaching following incorrect or absent motor preparation. Neuron 81, 438–451 (2014). 24462104 10.1016/j.neuron.2013.11.003 PMC3936035

[197] Elsayed GF & Cunningham J P Structure in neural population recordings: an expected byproduct of simpler phenomena? Nat. Neurosci. 20, 1310–1318 (2017). 28783140 10.1038/nn.4617 PMC5577566

[198] Li N, Daie K, Svoboda K & Druckmann S Robust neuronal dynamics in premotor cortex during motor planning. Nature 532, 459–464 (2016). 27074502 10.1038/nature17643 PMC5081260

[199] Geladi P & Kowalski BR Partial least-squares regression: a tutorial. Anal. Chim. Acta 185, 1–17 (1986).

[200] Remedios R Social behaviour shapes hypothalamic neural ensemble representations of conspecific sex. Nature 550, 388–392 (2017). 29052632 10.1038/nature23885 PMC5674977

[201] Kobak D Demixed principal component analysis of neural population data. eLife 5, 614–635 (2016). 10.7554/eLife.10989 PMC4887222 27067378

[202] Mante V, Sussillo D, Shenoy KV & Newsome WT Context-dependent computation by recurrent dynamics in prefrontal cortex. Nature 503, 78–84 (2013). 24201281 10.1038/nature12742 PMC4121670

[203] Williams AH Unsupervised discovery of demixed, low-dimensional neural dynamics across multiple timescales through tensor component analysis. Neuron 98, 1099–1115.e8 (2018). 29887338 10.1016/j.neuron.2018.05.015 PMC6907734

[204] Sani OG, Abbaspourazad H, Wong YT, Pesaran B & Shanechi MM Modeling behaviorally relevant neural dynamics enabled by preferential subspace identification. Nat. Neurosci. 24, 140–149. (2021). 33169030 10.1038/s41593-020-00733-0

[205] Sani OG, Pesaran B & Shanechi MM Where is all the nonlinearity: flexible nonlinear modeling of behaviorally relevant neural dynamics using recurrent neural networks. Preprint at bioRxiv 10.1101/2021.09.03.458628 (2021).

[206] Scangos KW, Makhoul GS, Sugrue LP, Chang EF & Krystal AD State-dependent responses to intracranial brain stimulation in a patient with depression. Nat. Med. 27, 229–231 (2021). 33462446 10.1038/s41591-020-01175-8 PMC8284979

[207] Semedo JD, Gokcen E, Machens CK, Kohn A & Yu BM Statistical methods for dissecting interactions between brain areas. Curr. Opin. Neurobiol. 65, 59–69 (2020). 33142111 10.1016/j.conb.2020.09.009 PMC7935404

[208] Kohn A Principles of corticocortical communication: proposed schemes and design considerations. Trends Neurosci. 43, 725–737 (2020). 32771224 10.1016/j.tins.2020.07.001 PMC7484382

[209] Hahn G, Ponce-Alvarez A, Deco G, Aertsen A & Kumar A Portraits of communication in neuronal networks. Nat. Rev. Neurosci. 20, 117–127 (2019). 30552403 10.1038/s41583-018-0094-0

[210] Zandvakili A & Kohn A Coordinated neuronal activity enhances corticocortical communication. Neuron 87, 827–839 (2015). 26291164 10.1016/j.neuron.2015.07.026 PMC4545497

[211] Buzsáki G & Wang X-J Mechanisms of gamma oscillations. Annu. Rev. Neurosci. 35, 203–225 (2012). 22443509 10.1146/annurev-neuro-062111-150444 PMC4049541

[212] Sohal VS, Zhang F, Yizhar O & Deisseroth K Parvalbumin neurons and gamma rhythms enhance cortical circuit performance. Nature 459, 698–702 (2009). 19396159 10.1038/nature07991 PMC3969859

[213] Kaufman MT, Churchland MM, Ryu SI & Shenoy KV Cortical activity in the null space: permitting preparation without movement. Nat. Neurosci. 17, 440–448 (2014). 24487233 10.1038/nn.3643 PMC3955357

[214] Semedo JD, Zandvakili A, Machens CK, Yu BM & Kohn A Cortical areas interact through a communication subspace. Neuron 102, 249–259.e4 (2019). 30770252 10.1016/j.neuron.2019.01.026 PMC6449210

[215] Keller EL Participation of medial pontine reticular formation in eye movement generation in monkey. J. Neurophysiol. 37, 316–332 (1974). 4205567 10.1152/jn.1974.37.2.316

[216] Kupfermann I & Weiss KR The command neuron concept. Behav. Brain Sci. 1, 3–10 (1978).

[217] Darlington TR & Lisberger SG Mechanisms that allow cortical preparatory activity without inappropriate movement. eLife 9, e50962 (2020). 32081130 10.7554/eLife.50962 PMC7060051

[218] Bassett DS & Sporns O Network neuroscience. Nat. Neurosci. 20, 353–364 (2017). 28230844 10.1038/nn.4502 PMC5485642

[219] Bassett DS, Zurn P & Gold JI On the nature and use of models in network neuroscience. Nat. Rev. Neurosci. 19, 566–578 (2018). 30002509 10.1038/s41583-018-0038-8 PMC6466618

[220] Xie ME High-fidelity estimates of spikes and subthreshold waveforms from 1-photon voltage imaging in vivo. Cell Rep. 35, 108954 (2021). 33826882 10.1016/j.celrep.2021.108954 PMC8095336

[221] Keshtkaran MR A large-scale neural network training framework for generalized estimation of single-trial population dynamics. Preprint at bioRxiv 10.1101/2021.01.13.426570 (2021). PMC9825111 36443486

[222] Pandarinath C Latent factors and dynamics in motor cortex and their application to brain-machine interfaces. J. Neurosci. 38, 9390–9401 (2018). 30381431 10.1523/JNEUROSCI.1669-18.2018 PMC6209846

[223] Pandarinath C Inferring single-trial neural population dynamics using sequential auto-encoders. Nat. Methods 15, 805–815 (2018). 30224673 10.1038/s41592-018-0109-9 PMC6380887

[224] Sylwestrak EL Cell type-specific population-dynamics of diverse reward computations. Cell 185, 3568–3587.e27 (2022). 36113428 10.1016/j.cell.2022.08.019 PMC10387374

[225] Aitken K The geometry of integration in text classification RNNs. Preprint at 10.48550/arXiv.2010.15114 (2020).

[226] Maheswaranathan N, Williams AH, Golub MD, Ganguli S & Sussillo D Universality and individuality in neural dynamics across large populations of recurrent networks. Preprint at 10.48550/arXiv.1907.08549 (2019). PMC7416639 32782422

[227] McIntosh L, Maheswaranathan N, Nayebi A, Ganguli S & Baccus S Deep learning models of the retinal response to natural scenes. Adv. Neural Inf. Process. Syst. 29, 1369–1377 (2016). 28729779 PMC5515384

[228] Sussillo D & Barak O Opening the black box: low-dimensional dynamics in high-dimensional recurrent neural networks. NeuralComput. 25, 626–649 (2013). 10.1162/NECO_a_00409 23272922

[229] Sauerbrei BA Cortical pattern generation during dexterous movement is input-driven. Nature 577, 386–391 (2019). 31875851 10.1038/s41586-019-1869-9 PMC6962553

[230] Perich MG Inferring brain-wide interactions using data-constrained recurrent neural network models. Preprint at bioRxiv 10.1101/2020.12.18.423348 (2021). PMC13565020 42567158

[231] Perich MG & Rajan K Rethinking brain-wide interactions through multi-region ‘network of networks’ models. Curr. Opin. Neurobiol. 65, 146–151 (2020). 33254073 10.1016/j.conb.2020.11.003 PMC7822595

[232] Lo C-C & Wang X-J Cortico-basal ganglia circuit mechanism for a decision threshold in reaction time tasks. Nat. Neurosci. 9, 956–963 (2006). 16767089 10.1038/nn1722

[233] Pinto L Task-dependent changes in the large-scale dynamics and necessity of cortical regions. Neuron 104, 810–824 (2019). 31564591 10.1016/j.neuron.2019.08.025 PMC7036751

[234] Hattori R & Komiyama T Context-dependent persistency as a coding mechanism for robust and widely distributed value coding. Neuron 10.1016/j.neuron.2021.11.001 (2021). PMC8813889 34818514

[235] Javadzadeh M & Hofer SB Dynamic causal communication channels between neocortical areas. Preprint at bioRxiv 10.1101/2021.06.28.449892 (2021). PMC9616801 35690063

[236] Gokcen E Disentangling the flow of signals between populations of neurons. Nat. Comput. Sci 2, 512–525 (2022). 10.1038/s43588-022-00282-5 PMC11442031 38177794

[237] Allen WE Thirst-associated preoptic neurons encode an aversive motivational drive. Science 357, 1149–1155 (2017). 28912243 10.1126/science.aan6747 PMC5723384

[238] Orsolic I, Rio M, Mrsic-Flogel TD & Znamenskiy P Mesoscale cortical dynamics reflect the interaction of sensory evidence and temporal expectation during perceptual decision-making. Neuron 109, 1861–1875.e10 (2021). 33861941 10.1016/j.neuron.2021.03.031 PMC8186564

[239] Xiao D Mapping cortical mesoscopic networks of single spiking cortical or sub-cortical neurons. eLife 6, e19976 (2017). 28160463 10.7554/eLife.19976 PMC5328594

[240] Carter ME Tuning arousal with optogenetic modulation of locus coeruleus neurons. Nat. Neurosci. 10.1038/nn.2682 (2010). PMC3174240 21037585

[241] Deisseroth K From microbial membrane proteins to the mysteries of emotion. Cell 184, 5279–5285 (2021). 34562367 10.1016/j.cell.2021.08.018 PMC10327439

[242] Cardin JA Functional flexibility in cortical circuits. Curr. Opin. Neurobiol. 58, 175–180 (2019). 31585330 10.1016/j.conb.2019.09.008 PMC6981226

[243] Gilad A, Gallero-Salas Y, Groos D & Helmchen F Behavioral strategy determines frontal or posterior location of short-term memory in neocortex. Neuron 99, 814–828.e7 (2018). 30100254 10.1016/j.neuron.2018.07.029

[244] Clancy KB & Mrsic-Flogel TD The sensory representation of causally controlled objects. Neuron 109, 677–689.e4 (2021). 33357383 10.1016/j.neuron.2020.12.001 PMC7889580

[245] Jacobs EAK, Steinmetz NA, Peters AJ, Carandini M & Harris KD Cortical state fluctuations during sensory decision making. Curr. Biol. 30, 4944–4955.e7 (2020). 33096037 10.1016/j.cub.2020.09.067 PMC7758730

[246] Colgin LL Oscillations and hippocampal-prefrontal synchrony. Curr. Opin. Neurobiol. 21, 467–474 (2011). 21571522 10.1016/j.conb.2011.04.006 PMC3578407

[247] Park AJ Reset of hippocampal–prefrontal circuitry facilitates learning. Nature 591, 615–619 (2021). 33627872 10.1038/s41586-021-03272-1 PMC7990705

[248] Sigurdsson T, Stark KL, Karayiorgou M, Gogos JA & Gordon JA Impaired hippocampal–prefrontal synchrony in a genetic mouse model of schizophrenia. Nature 464, 763–767 (2010). 20360742 10.1038/nature08855 PMC2864584

[249] Ferenczi EA Prefrontal cortical regulation of brainwide circuit dynamics and reward-related behavior. Science 10.1126/science.aac9698 (2016). PMC4772156 26722001

[250] Mimica B, Dunn BA, Tombaz T, Bojja VPTNCS & Whitlock JR Efficient cortical coding of 3D posture in freely behaving rats. Science 362, 584–589 (2018). 30385578 10.1126/science.aau2013

[251] Cramer JV In vivo widefield calcium imaging of the mouse cortex for analysis of network connectivity in health and brain disease. Neuroimage 199, 570–584 (2019). 31181333 10.1016/j.neuroimage.2019.06.014

[252] Hultman R Brain-wide electrical spatiotemporal dynamics encode depression vulnerability. Cell 173, 166–180.e14 (2018). 29502969 10.1016/j.cell.2018.02.012 PMC6005365

[253] Wang X Altered mGluR5-Homer scaffolds and corticostriatal connectivity in a Shank3 complete knockout model of autism. Nat. Commun. 7, 11459 (2016). 27161151 10.1038/ncomms11459 PMC4866051

[254] Buccino AP SpikeInterface, a unified framework for spike sorting. eLife 9, e61834 (2020). 33170122 10.7554/eLife.61834 PMC7704107

[255] Chung JE A fully automated approach to spike sorting. Neuron 95, 1381–1394.e6 (2017). 28910621 10.1016/j.neuron.2017.08.030 PMC5743236

[256] Lee J YASS: yet another spike sorter. Preprint at bioRxiv 10.1101/151928 (2017).

[257] Magland J SpikeForest, reproducible web-facing ground-truth validation of automated neural spike sorters. eLife 9, e55167 (2020). 32427564 10.7554/eLife.55167 PMC7237210

[258] Yger P A spike sorting toolbox for up to thousands of electrodes validated with ground truth recordings in vitro and in vivo. eLife 7, e34518 (2018). 29557782 10.7554/eLife.34518 PMC5897014

[259] Hazan L, Zugaro M & Buzsaki G Klusters, NeuroScope, NDManager: a free software suite for neurophysiological data processing and visualization. J. Neurosci. Methods 155, 207–216 (2006). 16580733 10.1016/j.jneumeth.2006.01.017

[260] Giovannucci A CalmAn an open source tool for scalable calcium imaging data analysis. eLife 8, e38173 (2019). 30652683 10.7554/eLife.38173 PMC6342523

[261] Mukamel EA, Nimmerjahn A & Schnitzer MJ Automated analysis of cellular signals from large-scale calcium imaging data. Neuron 63, 747–760 (2009). 19778505 10.1016/j.neuron.2009.08.009 PMC3282191

[262] Pachitariu M Suite2p: beyond 10,000 neurons with standard two-photon microscopy. Preprint at bioRxiv 10.1101/061507 (2017).

[263] Greenberg DS Accurate action potential inference from a calcium sensor protein through biophysical modeling. Preprint at bioRxiv 10.1101/479055 (2018).

[264] Pnevmatikakis EA, Merel J, Pakman A & Paninski L Bayesian spike inference from calcium imaging data. in Signals, Systems and Computers, 2013 Asilomar Conference on 349–353 (lEEE, 2013).

[265] Vogelstein J Fast nonnegative deconvolution for spike train inference from population calcium imaging. J. Neurophysiol. 104, 3691–3704 (2010). 20554834 10.1152/jn.01073.2009 PMC3007657

[266] Zhou P EASE: EM-assisted source extraction from calcium imaging data. Preprint at bioRxiv 10.1101/2020.03.25.007468 (2020).

[267] Berens P Community-based benchmarking improves spike rate inference from two-photon calcium imaging data. PLoS Comput. Biol. 14, e1006157 (2018). 29782491 10.1371/journal.pcbi.1006157 PMC5997358

[268] Rupprecht P A database and deep learning toolbox for noise-optimized, generalized spike inference from calcium imaging. Nat. Neurosci. 24, 1324–1337 (2021). 34341584 10.1038/s41593-021-00895-5 PMC7611618

[269] Song A, Gauthier JL, Pillow JW, Tank DW & Charles AS Neural anatomy and optical microscopy (NAOMi) simulation for evaluating calcium imaging methods. J. Neurosci. Methods 358, 109173 (2021). 33839190 10.1016/j.jneumeth.2021.109173 PMC8217135

[270] Zhou P Efficient and accurate extraction of in vivo calcium signals from microendoscopic video data. eLife 7, e28728 (2018). 29469809 10.7554/eLife.28728 PMC5871355

[271] Lu J MIN1PIPE: a miniscope 1-photon-based calcium imaging signal extraction pipeline. Cell Rep. 23, 3673–3684 (2018). 29925007 10.1016/j.celrep.2018.05.062 PMC6084484

[272] Friedrich J, Giovannucci A & Pnevmatikakis EA Online analysis of microendoscopic 1-photon calcium imaging data streams. PLoS Comput. Biol. 17, e1008565 (2021). 33507937 10.1371/journal.pcbi.1008565 PMC7842953

[273] Cardin JA Targeted optogenetic stimulation and recording of neurons in vivo using cell-type-specific expression of Channelrhodopsin-2. Nat. Protoc. 5, 247–254 (2010). 20134425 10.1038/nprot.2009.228 PMC3655719

[274] Kim K Artifact-free and high-temporal-resolution in vivo opto-electrophysiology with microLED optoelectrodes. Nat. Commun. 11, 2063 (2020). 32345971 10.1038/s41467-020-15769-w PMC7188816

[275] Chen R Deep brain optogenetics without intracranial surgery. Nat. Biotechnol. 39, 161–164 (2021). 33020604 10.1038/s41587-020-0679-9 PMC7878426

[276] Kishi KE Structural basis for channel conduction in the pump-like channelrhodopsin ChRmine. Cell 185, 672–689.e23 (2022). 35114111 10.1016/j.cell.2022.01.007 PMC7612760

[277] Inoue M Rational engineering of XCaMPs, a multicolor GECI suite for in vivo imaging of complex brain circuit dynamics. Cell 177, 1346–1360.e24 (2019). 31080068 10.1016/j.cell.2019.04.007

